# Targeting ROCK1/YAP1 Axis Ameliorates Inflammation‐Induced Prostatic Hyperplasia via Stabilising SIRT1‐Dependent Mitochondrial Dynamics

**DOI:** 10.1111/cpr.70085

**Published:** 2025-07-04

**Authors:** Dongxu Lin, Pengyu Wei, Mengyang Zhang, Kang Li, Lina Li, Zhipeng Li, Changcheng Luo, Wenbo Kuang, Kai Cui, Zhong Chen

**Affiliations:** ^1^ Department and Institute of Urology Tongji Hospital, Tongji Medical College, Huazhong University of Science and Technology Wuhan Hubei China; ^2^ Department of Rehabilitation Tongji Hospital, Tongji Medical College, Huazhong University of Science and Technology Wuhan Hubei China

**Keywords:** benign prostatic hyperplasia, inflammation, mitochondrial dynamics, SIRT1, YAP1

## Abstract

Benign prostatic hyperplasia (BPH) is a common condition in older men, with its prevalence increasing as age advances. Chronic inflammation orchestrates oxidative stress to exacerbate BPH. YAP1, which regulates organ size, cellular homeostasis, and tissue fibrosis, can be activated by ROCK1. Given the urgent clinical need for more effective therapies, this study explored whether targeting the ROCK1/YAP1 axis could mitigate BPH progression. Here, rats received in situ adeno‐associated virus (AAV) injection to induce prostate‐specific YAP1 overexpression. An inflammation‐associated experimental autoimmune prostatitis (EAP) model was established by prostate antigen immunisation, followed by treatment with ROCK1 inhibitor fasudil and YAP1 inhibitor verteporfin. Cell models were treated with specific inhibitors to confirm the critical role of YAP1 in modulating mitochondrial function. As a result, YAP1 overexpression was sufficient to induce a pathological BPH phenotype. Specifically, YAP1 activated the inflammatory cascade to provoke an immune response, disrupted proliferation/apoptosis balance to induce tissue hyperplasia, triggered epithelial‐mesenchymal transition (EMT) and reactive stroma to drive fibrosis, and promoted NOX4/ROS generation and antioxidant depletion to cause oxidative stress. The inflammation‐induced experimental autoimmune prostatitis (EAP) model also presented analogous BPH lesions, which were significantly alleviated when treated with ROCK1 inhibitor fasudil and YAP1 inhibitor verteporfin. Mechanistically, YAP1 activation under inflammatory conditions suppressed SIRT1 expression, thereby exacerbating oxidative stress through the disruption of DRP1/MFN2‐mediated mitochondrial dynamics. Overall, inflammation‐driven activation of the ROCK1/YAP1 axis aggravates oxidative stress, promoting BPH hyperplasia and fibrosis by impairing SIRT1‐regulated mitochondrial dynamics. These findings provide a preclinical rationale for developing ROCK1 or YAP1 inhibitors as targeted therapies for BPH patients with chronic inflammation.

## Graphical abstract

Prostate‐specific YAP1 overexpression induces pathological BPH phenogenesis. Targeting ROCK1/YAP1 with fasudil and verteporfin alleviates inflammatory BPH lesions. YAP1 orchestrates mitochondrial dysfunction and oxidative stress via suppressing SIRT1 function.

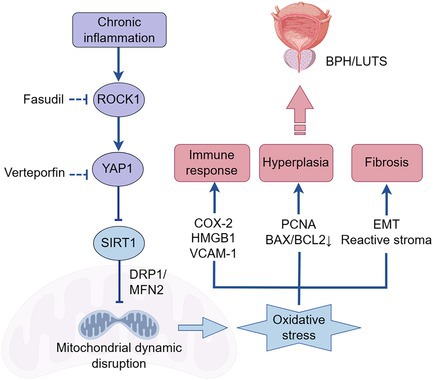

## 1 Introduction

Benign prostatic hyperplasia (BPH) is one of the predominant diseases afflicting elderly males, with its incidence increasing concomitantly with age. The prevalence of BPH is 50% in men over the age of 50, and it escalates to 80% in those over the age of 80 [1]. As screening methods spread and life expectancy extends, the incidence of BPH is expected to rise continuously. Histologically, BPH is characterised by the uncontrolled growth of epithelial and stromal components in the transition zone of the human prostate, leading to bladder outlet obstruction and subsequent lower urinary tract symptoms (LUTS).

Chronic inflammation exacerbates BPH progression, with prostatic inflammation leading to more severe LUTS and increased risk of urinary retention [2]. Combined therapy of the COX‐2 and 5α‐reductase inhibitors alleviated LUTS severity in BPH patients [3]. Patients with autoimmune diseases are more prone to BPH, and TNF‐α antagonists reduced prostatic inflammation and BPH incidence [4]. These findings suggest that autoimmune responses may disturb prostate immune homeostasis, thereby worsening BPH progression. Our previous studies showed that autoimmune inflammation could induce a rat BPH phenotype [5], while exogenous KLK1 administration prevented autoimmune inflammation‐caused BPH damage by improving microcirculation and inhibiting oxidative stress [6]. However, the exact mechanisms of prostatic inflammation‐induced oxidative stress remain unclear.

The Hippo pathway is a conserved signalling cascade, with its key effector, YAP1, acting as a transcriptional co‐activator that regulates processes like organ size, tissue regeneration, carcinogenesis, and fibrosis [7]. Despite its recognised roles, few studies have explored the role of YAP1 in BPH development. It has been reported that GPER inhibited YAP1 activation to maintain proliferation/apoptosis balance of prostatic epithelial cells in a Gαs/cAMP/PKA/LATS‐dependent manner [8]. Metformin activated AMPK to inhibit prostatic epithelial cell proliferation by preventing the AR‐mediated YAP1‐TEAD4 interaction [9]. Our previous in vitro experiments have demonstrated that inflammation and mechanical signals promoted prostate cell survival and fibrosis via the RhoA/ROCK1/YAP1 axis [10]. However, the therapeutic potential and underlying mechanisms of targeting the ROCK1/YAP1 axis in inflammation‐related BPH damage remain unresolved.

Sirtuin 1 (SIRT1), a NAD(+)‐dependent deacetylase, can maintain mitochondrial function by stabilising the mitochondrial fission/fusion balance [11]. Mitochondria are highly dynamic organelles that constantly undergo fusion and fission processes to modulate their morphology and dynamics. Dynamin‐related protein‐1 (DRP1) controls mitochondrial fission, while mitofusin 1 and 2 (MFN1 and MFN2) regulate outer membrane fusion, and optic atrophy‐1 (OPA1) governs inner membrane fusion [12]. Abnormal DRP1 activation and MFN2 deficiency will cause aberrant fission and defective fusion, generating a network of hyper‐fragmented mitochondria with fractured mitochondrial DNA and impaired oxidative phosphorylation [13]. The accumulated fragmented mitochondria generate free radicals, ultimately provoking oxidative stress [14].

Herein, in vivo models were employed to determine whether YAP1 activation can recapitulate BPH pathology and to evaluate the therapeutic effects of ROCK1/YAP1 inhibition by fasudil and verteporfin on inflammation‐driven prostatic lesions. As a result, prostate‐specific overexpression of YAP1 was sufficient to induce a BPH‐like phenotype in rats by enhancing inflammatory response, promoting prostatic hyperplasia and fibrosis, and provoking oxidative stress. Similarly, chronic inflammation induced analogous characteristic BPH pathological features. Notably, targeting the ROCK1/YAP1 axis with fasudil and verteporfin effectively reversed the inflammation‐caused BPH damage, potentially by restoring DRP1/MFN2‐associated mitochondrial dynamic balance in a SIRT1‐dependent manner.

## 2 Materials and Methods

### 2.1 Experimental Animals and Manipulation Procedures

Eight‐week‐old male Sprague Dawley rats were acclimated for one week in an SPF environment before the experiment. The animal study was aligned with the standards established by the NIH Guide for the Care and Use of Laboratory Animals (National Research Council). All animal experiments were approved and supervised by the Ethics Committee of the Experimental Animal Center of Tongji Hospital (TJH‐202301006). To assess YAP1 overexpression in BPH phenogenesis and the therapeutic potential of targeting the ROCK1/YAP1 axis, a two‐phase animal study was conducted. In the first period, twenty rats were divided into negative control (NC) and YAP1 overexpression (YAP1‐OE) groups. On days 1 and 28, either an empty vector or a YAP1 overexpression vector (synthesised by Genomeditech, Shanghai, China), dissolved in sterile PBS with 5% glycerol, was injected at multiple sites within the ventral, dorsolateral, and anterior lobes of the rat prostate. An equal amount of vector was administered to each rat. In the second period, thirty rats were allocated to five groups: Ctrl (sham + PBS), EAP (experimental autoimmune prostatitis model), EAP‐F (EAP + fasudil), EAP‐V (EAP + verteporfin), and EAP‐FV (EAP + fasudil + verteporfin). EAP was induced using prostate extract with complete Freund's adjuvant (CFA) on days 1 and 28 according to a previous protocol [6]. Drug administration began one day after the second immunisation. The five groups of rats received intraperitoneal administration every other day for 4 weeks with PBS (Ctrl), PBS (EAP), fasudil (EAP‐F, 15 mg/kg/day), verteporfin (EAP‐V, 10 mg/kg/day), or their combination (EAP‐FV). Rats were euthanized and tissue samples were collected at the end of treatment.

The ventral prostate lobes were paraffin‐embedded and processed for histopathological evaluation using routine stains (HE, Masson, toluidine blue) alongside immunostaining (IHC, IF). The dorsolateral and anterior lobes were snap‐frozen for downstream molecular and biochemical analyses, including WB, PCR, oxidative‐stress assays (MDA, GSH, SOD, CAT), ELISA, and ROS/TUNEL histochemical assays. Body and prostate weights were measured, and the prostate index was calculated using the formula: prostate index = prostate weight/body weight × 1000. All prostate lobes, including the ventral, dorsolateral, and anterior lobes, were weighed.

### 2.2 Cell Culture and Reagent Intervention

The human prostatic hyperplasia epithelial cell line BPH‐1 was acquired from Leibniz Institute DSMZ (Cat# ACC‐143, RRID:CVCL_1091) and cultured in RPMI‐1640 medium supplemented with 10% FBS. Subsequently, the cells were treated with the inflammation inducer lipopolysaccharide (LPS, 10 μM), the YAP1 inhibitor VP (2 μM), the SIRT1 inhibitor EX‐527 (5 μM), the DRP1 inhibitor Mdivi‐1 (5 μM) or the MFN activator MASM7 (2 μM) depending on specific research objectives (all purchased from MedChemExpress, NJ, USA). Specifically, LPS was dissolved in culture medium, whereas VP, EX‐527, Mdivi‐1, and MASM7 were dissolved in DMSO and diluted to the indicated concentrations prior to cell treatment.

### 2.3 Histological Examination

The prostate sections were stained with HE and Masson's trichrome dyes to observe histopathological features and collagen fibre density, respectively. Furthermore, toluidine blue dye was used to identify mast cells, with activated mast cells defined as those exhibiting dispersed metachromatic granules [15].

### 2.4 Histoscore Pathological Scoring System

The Histoscore protocol, comprehensively assessing epithelial morphology, matrix abundance, cell polarity, nuclear shape, and other architectural features, was employed to quantify the histopathological characteristics of the ventral lobe of the prostate [5]. The histopathological observations, considering both lesion severity and distribution pattern, were scored in a blinded manner.

### 2.5 Inflammatory Grade

Under 400× magnification, 5 random fields of HE‐stained ventral lobe sections were chosen to count inflammatory cells. The prostatic inflammatory lesions were graded by two researchers in a blinded manner based on a 4‐point grading scale [16]. Inflammatory grade was scored based on the following criteria. Grade I: ≤ 10 scattered inflammatory cells; Grade II: 11–20 clustered inflammatory cells without epithelial damage or lymphoid follicle formation; Grade III: > 20 clustered inflammatory cells with focal epithelial disruption or lymphoid follicle formation; and Grade IV: extensive inflammatory cell infiltration with significant epithelial destruction or lymphoid nodule/follicle formation.

### 2.6 Immunohistochemistry (IHC) and Immunofluorescence (IF)

For IHC analysis, prostate sections underwent deparaffinisation, rehydration, and antigen retrieval. After quenching endogenous peroxidase activity and blocking, sections were incubated with primary antibodies, followed by an HRP‐conjugated secondary antibody, DAB staining, and haematoxylin counterstaining.

For IF analysis, sections or cells were subjected to permeabilisation, washing, and blocking processes, followed by incubation with primary antibodies. Fluorescence‐labelled secondary antibodies and DAPI were used for protein and nuclei visualisation, respectively. Antibody details are in Table S1.

### 2.7 Western Blotting (WB)

Total proteins were extracted using RIPA buffer, while nuclear proteins were isolated using Nuclear‐Cytosol Extraction Kit (Applygen, Beijing, China). Briefly, Cytosol Extraction Buffers A and B were used to lyse the plasma membrane and remove cytosolic components, after which the nuclear membrane was broken with Nuclear Extraction Buffer to isolate nuclear proteins. The extracted proteins were separated on SDS‐PAGE gels and then transferred onto PVDF membranes. After blocking, the membranes were sequentially incubated with primary antibodies and HRP‐conjugated secondary antibodies. After treatment with ECL solution, the protein bands were exposed using the Bio‐Rad imager system. The antibody details are listed in Table S1.

### 2.8 Real‐Time PCR (RT‐PCR)

Total RNA was extracted using TRIzol reagent, and then reversely transcribed into cDNA applying PrimeScript RT Master Mix (Takara, Tokyo, Japan). The mRNA levels were evaluated using TB Green Premix Ex Taq II (Takara). Relative gene transcription levels were calculated employing the 2^‐△△Ct^ method, with *Gapdh* serving as the normalised control. The primer details are listed in Table S2.

### 2.9 Enzyme Linked Immunosorbent Assay (ELISA)

Serum samples were obtained from the carotid artery, while prostate tissue homogenates were prepared by mechanically homogenising the prostate with PBS. After centrifugation, the supernatant was collected to detect the concentrations of TNF‐α and IL‐1β using corresponding ELISA kits (ELK Biotechnology, Wuhan, China) following the manufacturer's protocols. Results were normalised to total protein concentration.

### 2.10 Cystometry

Under urethane anaesthesia, a PE‐50 catheter was inserted into the bladder through a dome incision and connected to an infusion pump and pressure transducer. Pressure signals were recorded using the BL‐420 N system (Techman Software, Chengdu, China). The urodynamic parameters were calculated as previously defined [17].

### 2.11 TUNEL Apoptosis Assay

Tissue sections were deparaffinised by xylene and rehydrated by graded alcohol. Apoptotic cells were identified using the One‐Step TUNEL In Situ Apoptosis Kit (Elabscience, Wuhan, China). The apoptosis index was measured using the following formula: apoptosis index = (number of positive cells/total number of epithelial cells) × 100.

### 2.12 Determination of ROS, MDA, GSH, SOD, CAT and Hyp Contents

Reactive oxygen species (ROS) in prostate frozen sections were detected using the 10 μM dihydroethidium (DHE; MedChemExpress) probe. An equal weight (100 mg) of prostatic sample was homogenised in 1000 μL of saline solution, and the supernatant was collected after centrifugation. The concentrations of malondialdehyde (MDA), glutathione (GSH), superoxide dismutase (SOD), catalase (CAT), as well as hydroxyproline (Hyp) in the supernatant were determined using respective detection kits (all purchased from Jiancheng Bioengineering Institute, Nanjing, China). Data were normalised to total protein concentration.

### 2.13 Flow Cytometry

ROS generation in BPH‐1 cells was assessed using flow cytometry. Cellular ROS were labelled with a DCFH‐DA probe (Elabscience), while the mitochondrial ROS were labelled with a MitoSOX Red probe (Thermo Fisher Scientific, MA, USA). The fluorescence intensity was measured by flow cytometry.

### 2.14 Mitochondrial Ultrastructure

Mitochondrial ultrastructure was visualised using MitoTracker staining and transmission electron microscopy (TEM). For MitoTracker staining, mitochondria were labelled with 100 nM MitoTracker Green (Thermo Fisher Scientific) and nuclei were stained with Hoechst 33342. For TEM analysis, cells were fixed in 2.5% glutaraldehyde, post‐fixed in 1% osmium tetroxide, dehydrated in ethanol and acetone, and embedded in epoxy resin. The tissue was cut into 60–80 nm thick sections, mounted on copper grids, and contrasted with 2% uranyl acetate and lead citrate before imaging under a transmission electron microscope (Hitachi HT7800, Tokyo, Japan).

### 2.15 Statistical Analysis

Statistical analyses and result visualisation were performed using GraphPad Prism 9 software. Results were presented as mean ± SD of at least three independent experiments. Differences between two groups were compared through either an unpaired Student's t‐test or Mann–Whitney U test, depending on data distribution. *p* < 0.05 was considered statistically significant.

## 3 Results

### 3.1 AAV‐Mediated YAP1 Overexpression Induced Prostate Hypertrophy

**FIGURE 1:**
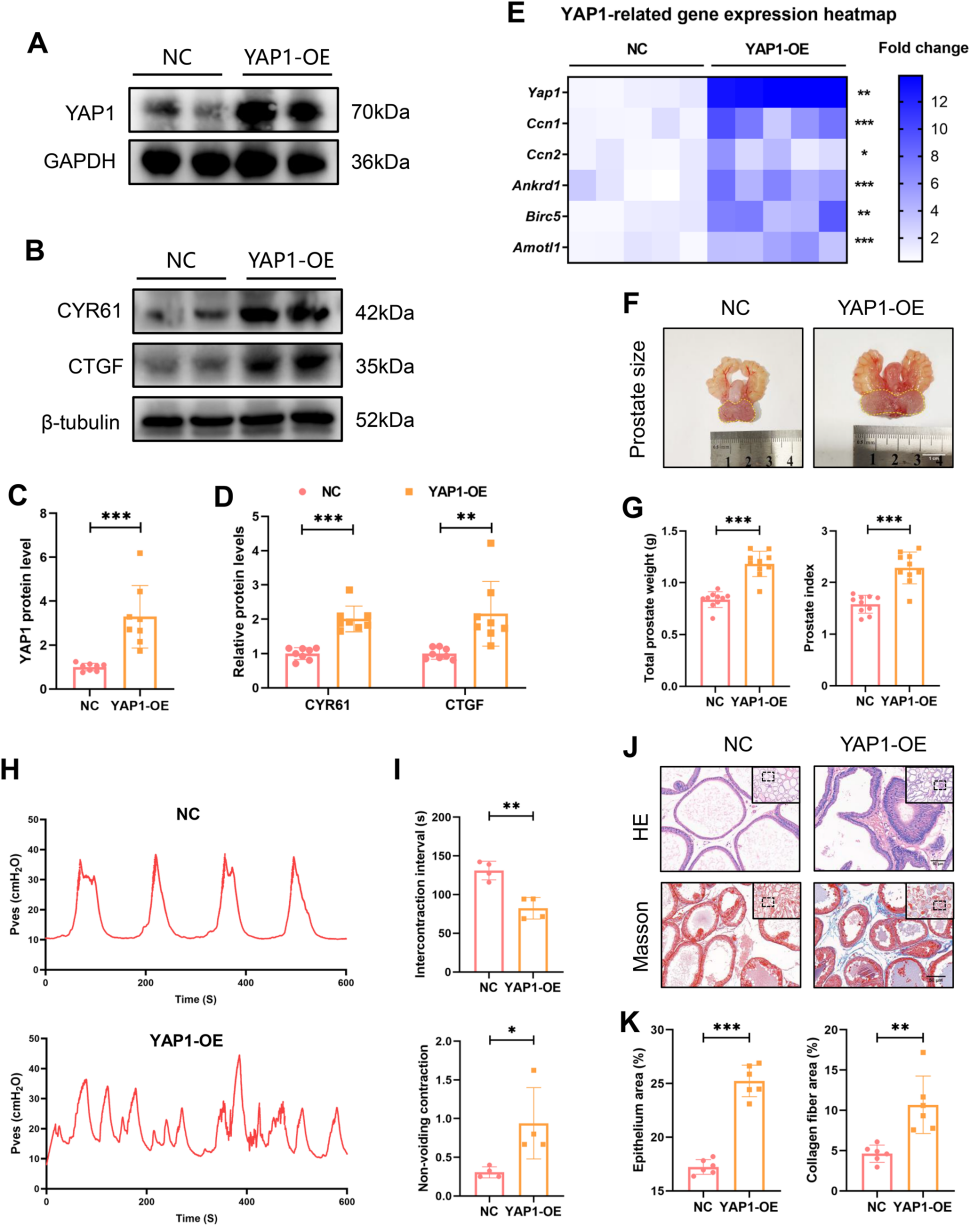
AAV‐mediated YAP1 overexpression resulted in prostate hypertrophy. (A‐D) WB analysis comparing the levels of YAP1 and YAP1‐regulated proteins (CYR61, CTGF) between the NC and YAP1‐OE groups. (E) RT‐PCR analysis of the mRNA levels of *Yap1* and its regulated genes (*Ccn1*, *Ccn2*, *Ankrd1*, *Birc5* and *Amotl1*), with results were visualzied as heatmap. (F, G) Representative photographs of prostate appearance, with the ventral lobe outlined by a dotted line, accompanied by comparisons of total prostate weight and prostate index between the two groups. (H, I) Representative intravesical pressure curves obtained by cystometry study, with comparisons of the intercontraction interval and non‐voiding contractions between the two groups. (J, K) Representative images of HE and Masson staining of the ventral lobe are presented, with evaluations performed on the areas of epithelium and collagen fibres.

To explore the detailed role of YAP1 in promoting BPH development, the rat prostate was injected in situ with a recombinant AAV vector to induce prostate‐specific *Yap1* overexpression. WB analysis confirmed that the successfully overexpressed YAP1 protein in the YAP1‐OE group led to a significant increase in the expression of its downstream tissue remodelling‐related proteins CYR61 and CTGF (Figure 1A–D). RT‐PCR results further demonstrated that the levels of *Yap1* and its downstream target genes (*Ccn1*, *Ccn2*, *Ankrd1*, *Birc5*, *Amotl1*) were significantly higher in the YAP1‐OE group compared to the NC group (Figure 1E), indicating successful enhancement of YAP1 expression and function in the YAP1‐OE group. YAP1 overexpression did not affect body weight but led to notable prostate enlargement, resulting in diffuse hypertrophy of the ventral, dorsolateral, and anterior lobes, and increased total prostate weight and prostate index (Figure 1F,G and Figure S1A–D). Cystometry analysis revealed that rats in the YAP1‐OE group exhibited bladder instability and dysfunction, characterised by a shorter voiding interval and more frequent non‐voiding contractions compared to those in the NC group (Figure 1H,I). Morphologically, the prostates in the NC group exhibited a uniform, monolayered cuboidal epithelium, whereas those in the YAP1‐OE group displayed an irregular, multilayered columnar epithelium with increased epithelial thickness and collagen fibre deposition (Figure 1J,K). Histoscore analysis revealed significantly higher total Histoscores in the YAP1‐OE group, indicative of pronounced pathological BPH characteristics (Table S3).

### 3.2 YAP1 Induced BPH Phenogenesis Through Aggravating Immune Response, Tissue Hyperplasia, and Fibrosis

**FIGURE 2:**
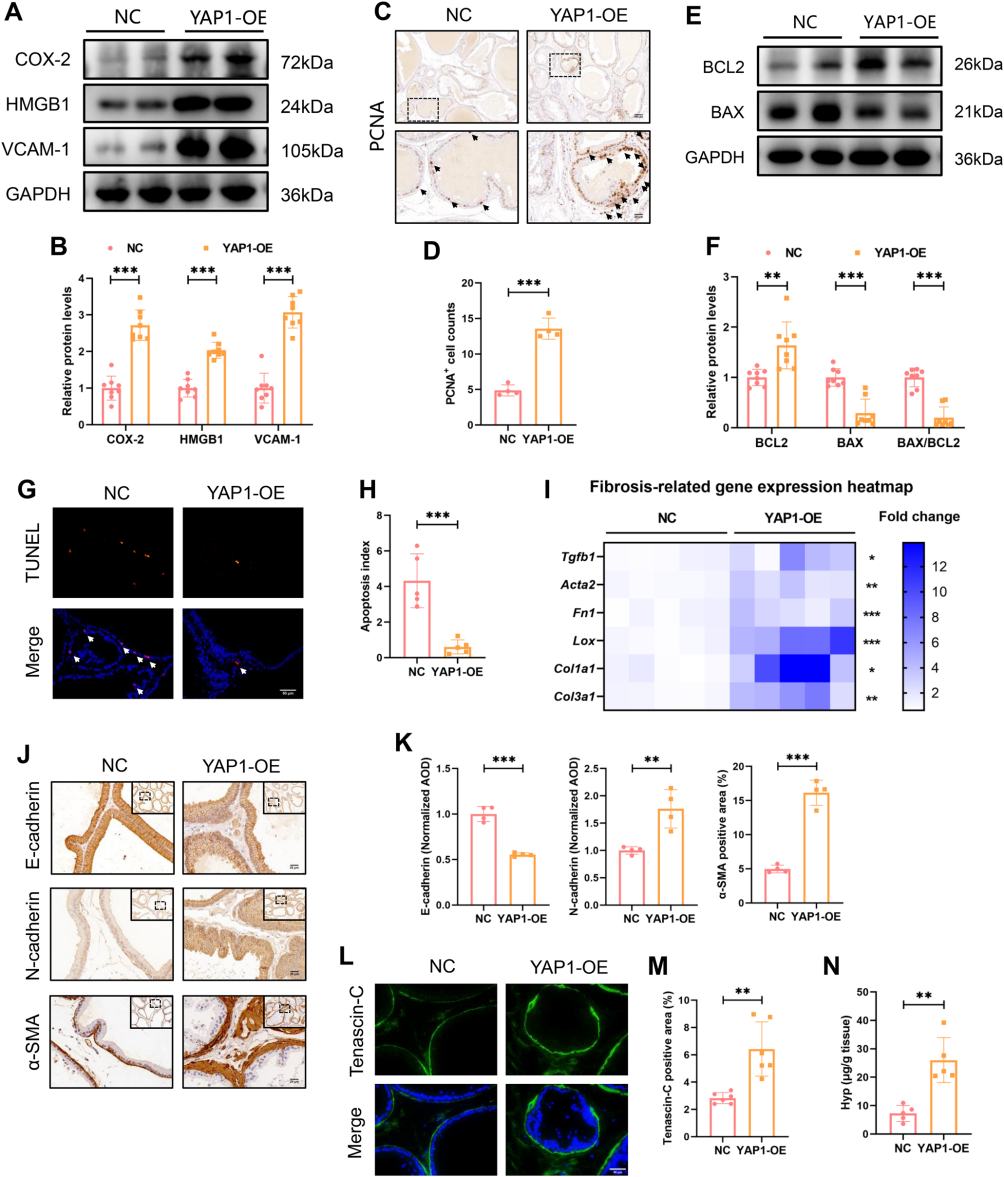
YAP1 induced BPH phenogenesis through aggravating immune response, tissue hyperplasia, and fibrosis. (A, B) WB analysis measuring the levels of proteins related to inflammatory cascades (COX‐2, HMGB1, VCAM‐1). (C, D) IHC analysis identifying PCNA‐reactive proliferating cells. (E, F) WB analysis determining the levels of apoptosis‐associated proteins (BCL2, BAX). (G, H) TUNEL staining was performed to identify apoptotic cells in each group. (I) RT‐PCR method analysing the mRNA expression of fibrosis‐associated genes (*Fn1*, *Tgfb1*, *Acta2*, *Col1a1*). ( J‐M ) IHC and IF methods analysing the expression of E‐cadherin, N‐cadherin, α‐SMA and Tenascin‐C to assess the occurrence of EMT and reactive stroma. (N) Comparison of the concentration of collagen biomarker Hyp between the two groups.

YAP1 overexpression amplified the inflammatory cascades by upregulating COX‐2, HMGB1, and VCAM‐1 expression (Figure 2A,B). Compared to the NC group, the YAP1‐OE group exhibited a dramatic increase in PCNA‐positive proliferating cells (Figure 2C,D), along with elevated levels of the anti‐apoptotic protein BCL2 and reduced levels of the pro‐apoptotic protein BAX (Figure 2E,F). The evidence of apoptosis resistance was further supported by a decrease in TUNEL‐positive apoptotic cells in the YAP1‐OE group (Figure 2G,H). These findings indicate that YAP1 activation is responsible for prostatic hyperplasia by promoting proliferation and inhibiting apoptosis. The mRNA levels of fibrosis‐associated genes, including *Tgfb1*, *Acta2*, *Fn1*, *Lox*, *Col1a1*, and *Col3a1*, were dramatically elevated in the YAP1‐OE group (Figure 2I). IHC analysis further revealed that YAP1 overexpression facilitated epithelial‐mesenchymal transition (EMT) and reactive stroma formation, as evidenced by the loss of the epithelial marker E‐cadherin and the gain of the mesenchymal marker N‐cadherin, and an increased α‐SMA‐positive stromal compartment along with elevated Tenascin‐C expression (Figure 2J–M). The content of collagen biomarker Hyp was also increased in the YAP1‐OE group (Figure 2N), further demonstrating a pro‐fibrogenic role of YAP1 in BPH development.

### 3.3 YAP1 Overexpression Provoked Oxidative Stress via Impairing Mitochondrial Dynamic Balance

**FIGURE 3:**
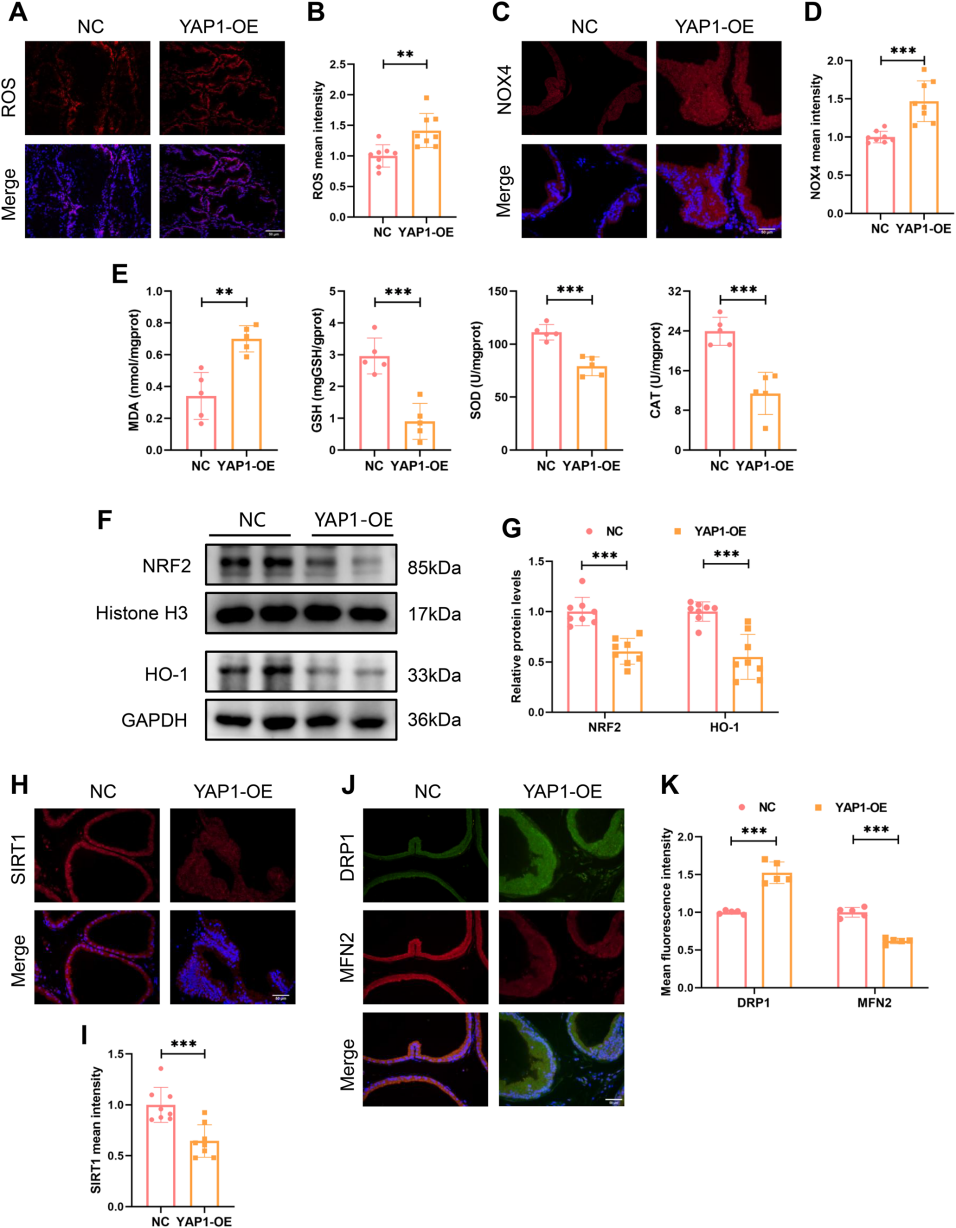
YAP1 overexpression provoked oxidative stress via impairing mitochondrial dynamic balance. (A, B) Fluorometric analysis of ROS abundance using the DHE probe. (C, D) IF analysis determining NOX4 expression in the prostates of the two groups. (E) Determination of MDA, GSH, SOD and CAT concentrations in prostate homogenates. (F, G) Representative immunoblot bands comparing the expression of NRF2 (nuclear) and HO‐1 (total) between the two groups. (H, I) IF analysis measuring SIRT1 expression in both groups. (J, K) IF analysis measuring DRP1 and MFN2 expression in both groups.

Previous studies have highlighted the pivotal role of the YAP1/ROS signalling in promoting oxidative stress [18]. In the YAP1‐OE group, the prostate tissue demonstrated a significantly higher degree of ROS accumulation (Figure 3A,B), and increased levels of the ROS‐generating enzyme NOX4 (Figure 3C,D). Additionally, there was an increase in the lipid peroxidation byproduct MDA, and a decrease in antioxidants such as GSH, SOD, and CAT in the YAP1‐OE group (Figure 3E). IF analysis showed a downregulation of NRF2 and HO‐1 proteins in the YAP1‐OE group (Figure S2A–B), a result further validated by WB analysis, which showed reduced nuclear NRF2 and total HO‐1 levels (Figure 3F,G).

The underlying mechanisms of YAP1‐mediated oxidative stress were further elucidated. Emerging evidence has demonstrated that SIRT1 can improve mitochondrial function, and thus mitigate oxidative damage, inflammation and fibrosis [19, 20]. The present study found that YAP1 overexpression significantly reduced the expression of SIRT1 (Figure 3H,I). Moreover, YAP1 overexpression resulted in the disruption of mitochondrial fission/fusion balance, characterised by the upregulation of DRP1 and the downregulation of MFN2 (Figure 3J,K). These findings suggest that YAP1 may trigger oxidative stress by inhibiting SIRT1 expression and impairing mitochondrial dynamic homeostasis.

### 3.4 Targeting the ROCK1/YAP1 Axis with Fasudil and Verteporfin Ameliorated Inflammation‐Induced Prostate Hypertrophy and Immune Responses

**FIGURE 4:**
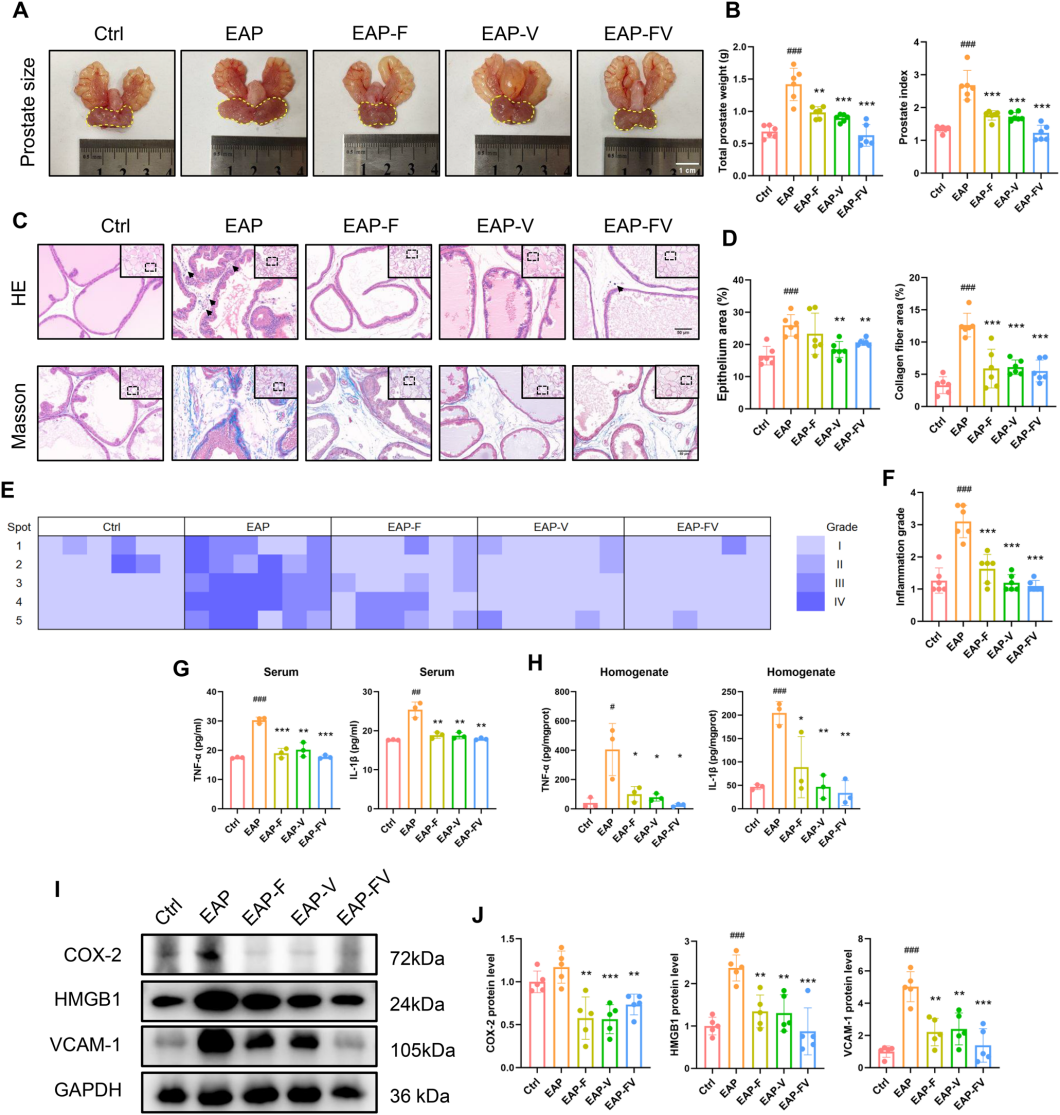
Targeting the ROCK1/YAP1 axis ameliorated inflammation‐induced prostate hypertrophy and immune responses. (A, B) Representative photographs of prostate morphology, with the ventral lobe outlined by a dotted line, accompanied by comparisons of total prostate weight and prostate index among different groups. (C, D) Representative images of HE staining and Masson staining, along with comparisons of epithelial and collagen fibre areas among different groups. Arrows indicate inflammatory cells. (E) The inflammatory grade of prostate lesion was scored and visualised as a heatmap. The columns represent 5 random spots within one section, while the rows denote 6 rats per group. (F) Quantitative analysis of inflammatory grade. (G, H) ELISA assay measuring the concentrations of proinflammatory cytokines (TNF‐α, IL‐1β) in serum and prostatic homogenates, respectively. (I, J) WB analysis examining the protein levels of inflammation‐associated molecules (COX‐2, HMGB1, VCAM‐1).

Extracellular matrix stiffness and inflammatory stimuli can activate the RhoA/ROCK1 pathway to modulate YAP activity [10, 21]. Here, we investigated the therapeutic potential of the clinically used ROCK inhibitor fasudil and YAP1 inhibitor verteporfin in treating inflammation‐induced BPH lesions. WB and IF analyses showed higher ROCK1 and YAP1 expression in the EAP model compared to the Ctrl group, which was effectively suppressed by treatment with fasudil (EAP‐F), verteporfin (EAP‐V), or their combination (EAP‐FV) (Figure S3A–D). These treatments also alleviated prostatic hypertrophy, as evidenced by a significant reductions in the weights of the ventral, dorsolateral, and anterior lobes, resulting in decreased total prostate weight and index (Figure 4A,B and Figure S4A–D). Histologically, epithelial stacking, stromal collagen fibre deposition and inflammatory cell infiltration were observed in the prostate from the EAP group, whereas these phenomena were dramatically alleviated following fasudil and verteporfin treatment (Figure 4C,D).

The inflammatory grade was scored to assess the severity of inflammation among the different groups. The heatmap indicated significant inflammatory lesions in the prostate from the EAP group, which were markedly alleviated after administration of fasudil and verteporfin (Figure 4E,F). Targeting the ROCK1/YAP1 axis with fasudil and verteporfin also decreased the production of pro‐inflammatory cytokines (TNF‐α and IL‐1β) in both the serum and prostate homogenates (Figure 4G,H). Additionally, both drugs prevented the infiltration of CD4^+^ T helper cells, CD8^+^ T cytotoxic cells, and CD68^+^ macrophages in the prostate microenvironment (Figure S5A–F). In the EAP group, mast cells were recruited to the prostate and degranulated to release inflammatory mediators, which was mitigated with fasudil and verteporfin treatment (Figure S6A–B). Both drugs, alone or in combination, significantly inhibited the expression of inflammatory signatures such as COX‐2, HMGB1, VCAM‐1 (Figure 4I,J).

### 3.5 Targeting the ROCK1/YAP1 Axis Alleviated Inflammation‐Induced Prostatic Tissue Hyperplasia and Fibrosis

**FIGURE 5:**
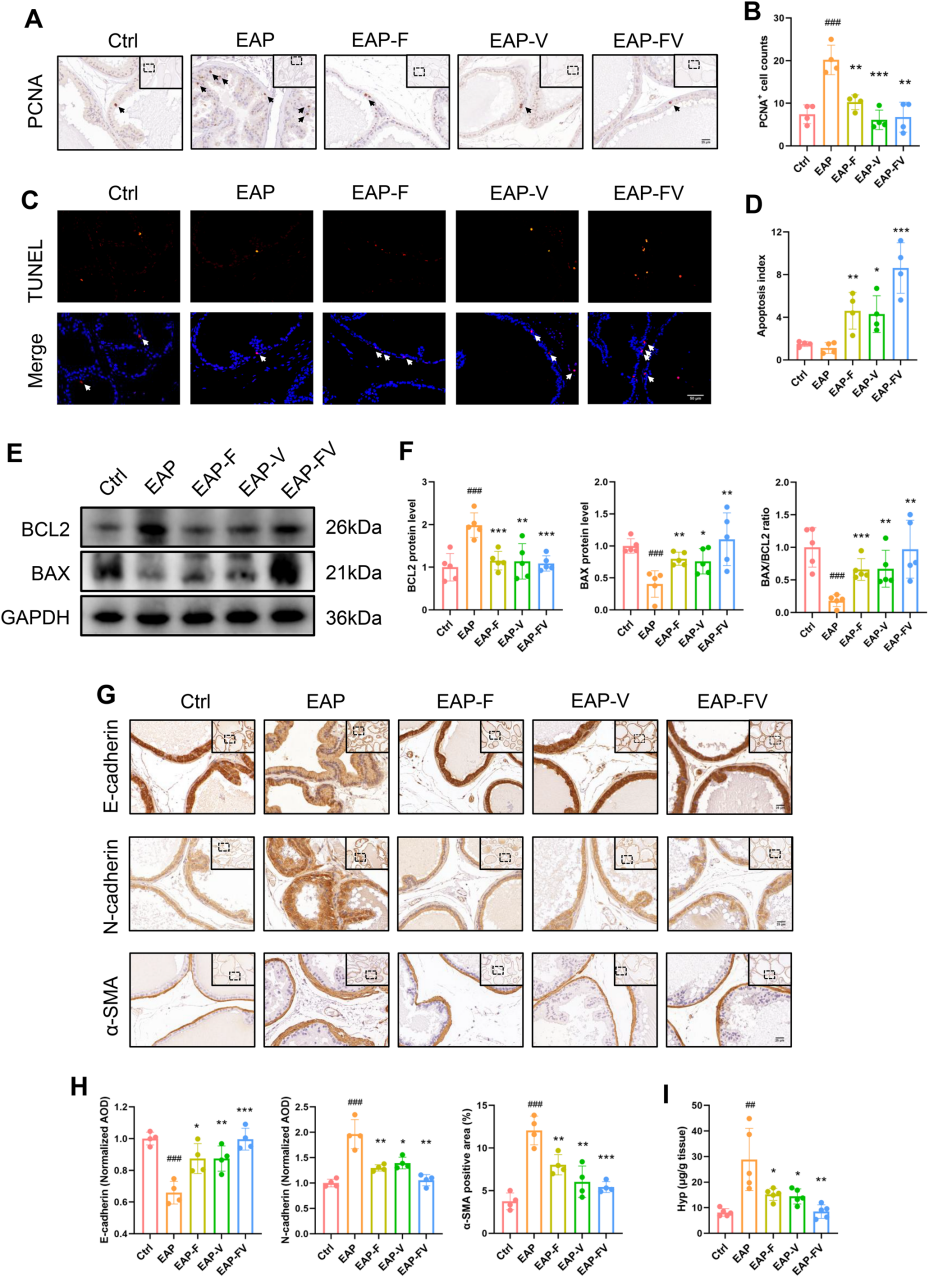
Targeting the ROCK1/YAP1 axis alleviated inflammation‐induced prostatic tissue hyperplasia and fibrosis. (A, B) IHC analysis identifying PCNA‐positive cells to assess proliferating capacity. (C, D) TUNEL staining to recognise apoptotic cells among different groups. (E, F) WB method comparing the levels of apoptosis‐associated proteins (BCL2, BAX). (G, H) IHC method analysing the expression of E‐cadherin, N‐cadherin, and α‐SMA to detect the occurrence of EMT and reactive stroma. (I) The Hyp content, which reflects the degree of fibrosis, was examined via a specific assay.

Inhibiting the ROCK1/YAP1 axis with fasudil and verteporfin significantly reversed inflammation‐induced prostatic tissue hyperplasia, as evidenced by fewer PCNA‐positive proliferating cells (Figure 5A,B), and more TUNEL‐positive apoptotic cells in the drug‐treated groups (Figure 5C,D). Meanwhile, inhibiting the ROCK1/YAP1 axis protected against inflammation‐triggered apoptosis resistance by inducing BAX expression and reducing BCL2 expression, which resulted in an increased BAX/BCL2 ratio (Figure 5E,F). Moreover, in the EAP group, the prostate underwent an EMT switch and reactive stroma formation, as indicated by decreased E‐cadherin expression and increased expression of N‐cadherin, α‐SMA and Tenascin‐C. However, these changes were reversed in the presence of fasudil and verteporfin (Figure 5G,H and Figure S7A–B). These drugs also reduced inflammation‐caused Hyp generation (Figure 5I), indicating fibrosis mitigation after ROCK1/YAP1 inhibition.

### 3.6 Inhibition of ROCK1/YAP1 Pathway Reversed Prostatic Inflammation‐Induced Oxidative Stress Damage

**FIGURE 6:**
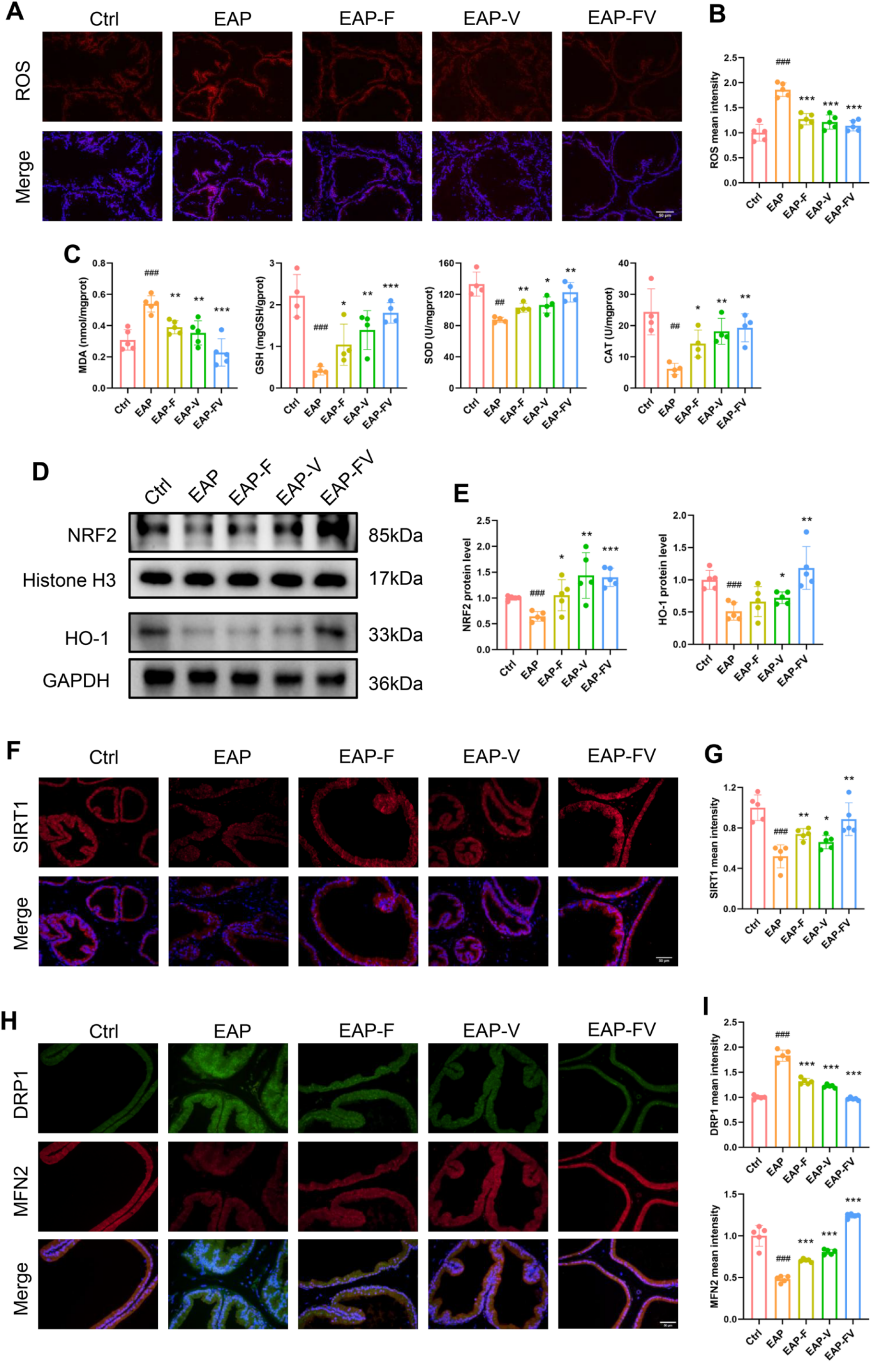
Inhibition of the ROCK1/YAP1 pathway reversed prostatic inflammation‐induced oxidative stress damage. (A, B) DHE fluorescent probe was used to determine the abundance of ROS in each group. (C) Concentrations of MDA, GSH, SOD, and CAT in prostate homogenates were measured using corresponding commercial kits. (D, E) Representative immunoblot bands to evaluate the expression of NRF2 (nucleus) and HO‐1 (total) in each group. (F, G) Representative fluorescent images and quantitative analysis of SIRT1 in each group. (H, I) Representative fluorescent images and quantitative analysis of DRP1 and MFN2 expression in each group.

Chronic inflammation can disturb redox homeostasis, which leads to DNA damage and genetic mutations, thereby worsening BPH progression [5]. This study revealed that inflammation triggered intracellular ROS synthesis partly due to elevation of NOX4, while inhibiting the ROCK1/YAP1 axis with fasudil and verteporfin effectively reduced NOX4‐induced ROS synthesis (Figure 6A,B and Figure S8A–B). Furthermore, the drug treatment groups exhibited a substantial decrease in the lipid peroxidation product MDA level, along with a significant increase in antioxidants (GSH, SOD, CAT) levels (Figure 6C). Meanwhile, targeting the ROCK1/YAP1 pathway reactivated the NRF2/HO‐1 antioxidant defence system (Figure 6D,E and Figure S9A–B). Furthermore, targeting the ROCK1/YAP1 axis contributed to the elevation of SIRT1 expression, which was accompanied by the downregulation of DRP1 and the upregulation of MFN2 (Figure 6F–I).

### 3.7 Inflammation‐Induced YAP1 Activation Suppressed SIRT1 Function to Impair Mitochondrial Dynamics

**FIGURE 7:**
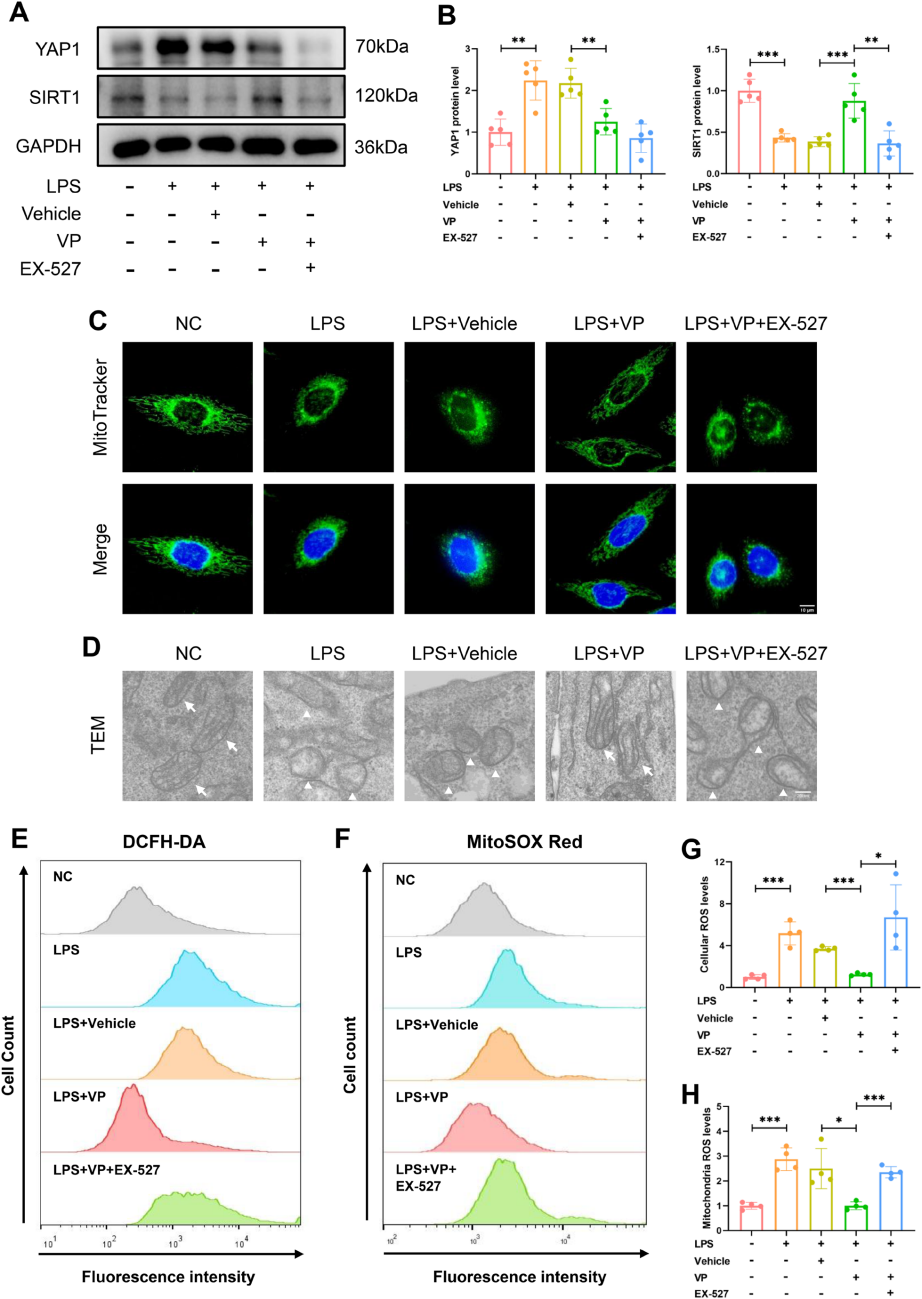
Inflammation‐induced YAP1 activation suppressed SIRT1 function to impair mitochondrial function. (A, B) Immunoblot analysis showing the inhibitory effects of VP and EX‐527 on YAP1 and SIRT1 expression. (C) MitoTracker Green staining was used to observe the mitochondrial morphology. (D) The morphological ultrastructures of mitochondria were evaluated by TEM. (E‐H) Flow cytometry was performed to evaluate the cellular and mitochondrial ROS levels using DCFH‐DA dye and MitoSOX Red dye, respectively.

To ascertain the hypothesis that YAP1 provokes prostatic oxidative stress via disruption of SIRT1‐dependent mitochondrial dynamics, we treated the human prostatic hyperplasia epithelial cell line BPH‐1 with the inflammation inducer LPS, the YAP1 inhibitor verteporfin (VP) and the SIRT1 inhibitor EX‐527 (Figure 7A,B). The results showed that the YAP1‐mediated inhibition of SIRT1 function was responsible for the suppression of the NRF2/HO‐1 antioxidant defence system (Figure S10A–B). MitoTracker staining revealed that VP treatment prevented LPS‐induced mitochondrial fragmentation and restored the mitochondrial network, while the addition of EX‐527 induced a marked increase in mitochondrial fragmentation and branch shortening (Figure 7C). TEM analysis further demonstrated that LPS‐treated groups exhibited swollen mitochondria with reduced or absent cristae structures compared to the NC group. However, inflammation‐caused mitochondrial damage was alleviated after YAP1 inhibition but worsened again following SIRT1 inhibition (Figure 7D). DCFH‐DA and MitoSOX Red staining demonstrated that LPS elevated both cellular and mitochondrial ROS production, which was diminished by VP but restored by EX‐527 (Figure 7E–H). These results suggest that inflammation‐activated YAP1 triggers oxidative stress through inhibiting SIRT1 function.

### 3.8 Mitochondrial Dynamics Mediated by DRP1 and MFN2 Affected BPH Progression

**FIGURE 8:**
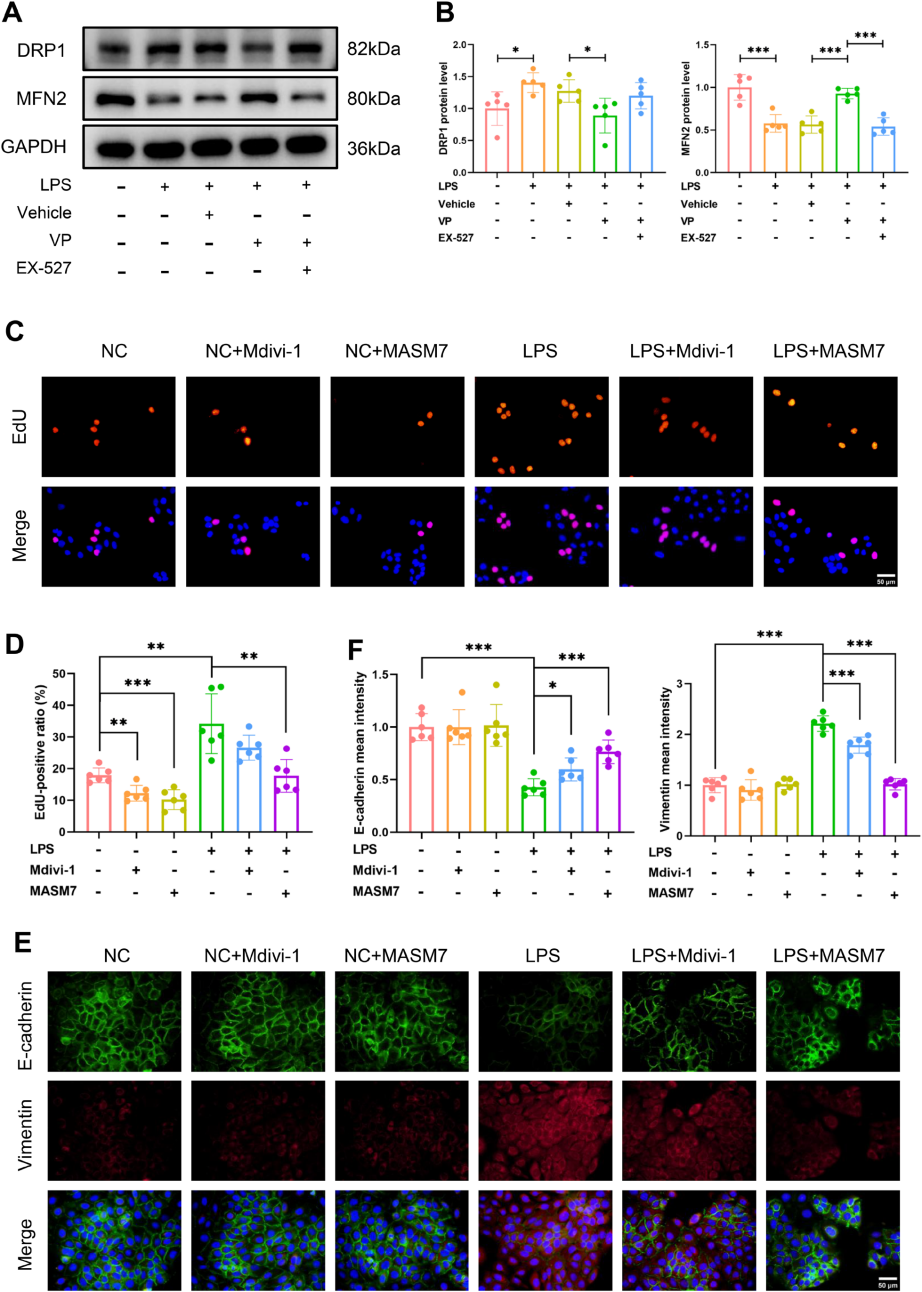
Mitochondrial dynamics mediated by DRP1 and MFN2 affected BPH progression. (A, B) Immunoblot analysis determining the regulatory effects of the YAP1‐SIRT1 axis on DRP1 and MFN2 expression. (C, D) IF analysis displaying the EdU‐positive proliferating cells. (E, F) IF analysis assessing the occurrence of EMT switch by determining E‐cadherin and Vimentin expression.

WB results further unveiled that YAP1‐mediated SIRT1 suppression was responsible for the increase in the mitochondrial fission protein DRP1 and the decrease in the fusion protein MFN2 (Figure 8A,B). Subsequently, the DRP1 antagonist Mdivi‐1 and the MFN2 agonist MASM7 were applied to ascertain the role of mitochondrial dynamics homeostasis in BPH progression. As a result, DRP1 inhibition and MFN2 activation significantly impeded the proliferative processes elicited by LPS, both of which manifested as reduced proportions of EdU‐positive proliferative cells (Figure 8C,D). They also reversed the LPS‐triggered EMT switch, characterised by the restoration of E‐cadherin and the loss of Vimentin (Figure 8E,F). Intriguingly, both Mdivi‐1 and MASM7 were able to mitigate the progression of inflammation‐driven BPH, albeit with a more pronounced effect in the MASM7‐treated group. Collectively, these findings suggest that inflammation‐induced YAP1 activation inhibits SIRT1 expression, disrupting mitochondrial dynamics primarily through the suppression of MFN2‐mediated mitochondrial fusion.

## 4 Discussion

BPH is characterised by uncontrolled hypertrophy in the transition zone of the human prostate. YAP1, a key effector of the Hippo pathway, has garnered wide interest due to its significant role in controlling organ size and fibrosis [7]. For instance, pressure overload promoted the interaction of YAP1 with TEAD1 and HIF‐1α and thus activated the GLUT1‐associated Warburg effect to induce compensatory myocardial hypertrophy [22]. Upon high glucose stress, YAP1 caused myocardial hypertrophy and fibrosis via activating the Akt/FOXM1 axis [23]. CAPZ depletion reinforced tissue mechanical properties, activating YAP1 to induce liver organ overgrowth, which could be rescued by the ROCK inhibitor fasudil [24]. The present study demonstrated that AAV‐induced YAP1 overexpression contributed to pathological changes indicative of prostatic hypertrophy, accompanied by strengthened immune responses, proliferation/apoptosis imbalance, and fibrosis, suggesting that YAP1 provokes the proliferative potential of prostatic cells to induce BPH phenogenesis.

Fasudil, the only clinically approved ROCK inhibitor, has been used for conditions like cerebral vasospasm and stable angina [25, 26]. Furthermore, fasudil activated NRF2 to counteract oxidative stress and inhibit the EMT process in hyperuricemic nephropathy [27]. Fasudil also induced M2 macrophage polarisation to reduce inflammation in autoimmune encephalomyelitis [28]. These studies suggest that fasudil holds significant therapeutic value in alleviating oxidative damage, immune responses, and organ fibrosis. Verteporfin has been clinically applied in the treatment of macular degeneration‐associated ocular vascular diseases [29]. Recent evidence has demonstrated that verteporfin specifically inhibited YAP1 activity by blocking the YAP1‐TEAD interaction [30]. By antagonising YAP1 activity, verteporfin exerted synergistic anti‐proliferative and anti‐invasive effects in preclinical glioblastoma models, resulting in improved survival outcomes [31]. Targeting YAP1 with verteporfin‐loaded biomimetic nanoparticles disrupted synoviocyte glycolysis and prevented M1 macrophage infiltration, thereby inhibiting diabetic osteoarthritis progression [32]. The activation of the ROCK1/YAP1 axis was responsible for colitis‐associated intestinal obstruction due to its pro‐fibrogenic effects [21]. Extracellular matrix stiffness promoted YAP1 activation to maintain the cancer‐associated fibroblast phenotype, which could be reversed by ROCK inhibition [33]. In this study, blocking the ROCK1/YAP1 axis with fasudil and verteporfin significantly attenuated prostate lesions caused by autoimmunity‐induced inflammation, manifested as a reduction in the inflammatory response, proliferation and fibrosis, as well as promotion of apoptosis. These findings provide proof‐of‐concept evidence supporting the blockade of the ROCK1/YAP1 axis with fasudil and verteporfin for treating inflammation‐associated BPH damage.

The rapid cell turnover rate and the relative lack of DNA repair enzymes in human prostate tissue make the prostate particularly vulnerable to oxidative stress damage. Chronic inflammation results in excessive ROS production and antioxidant depletion, thereby initiating oxidative stress and exacerbating BPH progression [34]. Emerging evidence indicates that YAP1 can modulate mitochondrial function and thus aggravate oxidative stress. YAP1 overexpression induced myocardial hypertrophy by impairing DNM1L/MFN1‐related mitochondrial function, while verteporfin prevented mitochondrial dysfunction and pathological hypertrophy [35]. Quercetin ameliorated high glucose‐induced mesangial cell proliferation, inflammation, and oxidative stress by modulating the miR‐485‐5p/YAP1 axis [36]. In our experiments, YAP1 triggered lipid peroxidation and disrupted antioxidant defence, as evidenced by the accumulation of MDA and the depletion of GSH, SOD, and CAT, which might be attributed to the suppression of NRF2/HO‐1 antioxidant signalling. Disruption of redox homeostasis driven by YAP1 overexpression resulted in excessive ROS accumulation, causing abnormal oxidative damage in the prostate.

SIRT1 regulates various cellular processes such as mitochondrial biogenesis, metabolic regulation, and antioxidant defence. Growing evidence has proved that SIRT1 decreased DRP1 expression and increased MFN2 expression to maintain mitochondrial dynamic balance and redox homeostasis [37, 38]. DRP1 deficiency and/or reconstituted MFN2 expression stabilised the mitochondrial fusion/fission dynamic balance to maintain mitochondrial function, which in turn alleviated oxidative stress damage [39, 40]. Here, blocking the ROCK1/YAP1 axis increased the levels of endogenous free radical scavengers (GSH, SOD, and CAT), activated the NRF2/HO‐1 antioxidant defence system and thus reduced the generation of NOX4/ROS oxidants to prevent inflammation‐triggered oxidative stress. Mechanistically, inflammation‐induced activation of YAP1 repressed SIRT1 expression, thereby exacerbating oxidative stress through disruption of DRP1/MFN2‐mediated mitochondrial dynamics. These findings imply that YAP1 serves as a critical orchestrator of inflammation‐caused prostatic oxidative stress, driven by mitochondrial dysfunction and impaired antioxidant defences due to SIRT1 inhibition.

However, this study also has several limitations. First, although prostate‐specific YAP1 overexpression provokes bladder instability, future studies should establish whether ROCK1/YAP1 inhibitors can restore bladder function in the EAP model. Second, although gross inspection and prostate weighing indicated uniform enlargement of the prostate in both the YAP1‐OE and EAP groups compared to the control group, comprehensive evaluation of all prostate lobes in further experiments will be essential to fully characterise lobe‐specific changes.

## 5 Conclusions

This study provides a novel perspective on the regulation of YAP1‐mediated redox homeostasis during BPH progression. Specifically, inflammatory stimulation promotes ROCK1 and YAP1 expression to exacerbate oxidative stress, which likely occurs through disrupting fusion/fission balance mediated by DRP1/MFN2 in a SIRT1‐dependent manner. By restoring SIRT1 expression, the ROCK1 kinase inhibitor fasudil and the YAP1 inhibitor verteporfin maintain mitochondrial dynamics and thus mitigate oxidative stress. This, in turn, prevents excessive immune responses, proliferation/apoptosis imbalance, and fibrotic reactions. Targeting the ROCK1/YAP1 pathway with fasudil and verteporfin may represent a feasible therapeutic strategy for BPH patients troubled by chronic inflammation.

## Author Contributions

Dongxu Lin: Conceptualization, Methodology, Investigation, Funding acquisition, Writing – original draft. Pengyu Wei, Mengyang Zhang, Kang Li, and Lina Li: Formal analysis, Visualisation, Investigation. Zhipeng Li, Changcheng Luo, and Wenbo Kuang: Project administration, Validation, Writing – Reviewing and Editing. Kai Cui, and Zhong Chen: Conceptualization, Supervision, Funding acquisition. All authors have read and approved the final manuscript.

## Ethics Statement

All animal experiments were approved and supervised by the Ethics Committee of the Experimental Animal Center of Tongji Hospital (TJH‐202301006).

## Conflicts of Interest

The authors declare no conflicts of interest.

## Supporting information


**Data S1.** Supporting Information.

## Data Availability

The data that support the findings of this study are available from the corresponding author upon reasonable request.
