# Supplementary material for: Targeting ROCK1/YAP1 Axis Ameliorates Inflammation‐Induced Prostatic Hyperplasia via Stabilising SIRT1‐Dependent Mitochondrial Dynamics
Source: Cell Prolif. 2025 Jul 4;59(2):e70085. doi: 10.1111/cpr.70085 (PMC12877945; doi:10.1111/cpr.70085)
Supplement: Supplementary file 1 — Data S1. Supporting Information. [file CPR-59-e70085-s001.pdf]

Supplementary Materials

Targeting ROCK1/YAP1 Axis Ameliorates Inflammation-Induced Prostatic Hyperplasia via Stabilizing SIRT1-Dependent Mitochondrial Dynamics

TABLE S1. The information of primary antibodies used in this study.

TABLE S2. The sequences of RT-PCR primers used in this study.

TABLE S3. Evaluating the prostatic pathological alterations using the Histoscore protocol.

FIGURE S1. YAP1 overexpression induced diffuse enlargement of all prostate lobes in rats.

FIGURE S2. YAP1 overexpression suppressed NRF2/HO-1 antioxidant defense system.

FIGURE S3. Fasudil and verteporfin were sufficient to inhibit inflammation-induced prostatic ROCK1 and YAP1 expression.

FIGURE S4. Targeting the ROCK1/YAP1 pathway attenuated inflammation-induced enlargement of various prostate lobes.

FIGURE S5. Inhibition of the ROCK1/YAP1 axis prevented inflammatory cells infiltration in prostate.

FIGURE S6. Inhibition of the ROCK1/YAP1 axis decreased mast cell accumulation and activation.

FIGURE S7. Inhibition of the ROCK1/YAP1 pathway reduced inflammation-induced Tenascin-C expression.

FIGURE S8. Suppression of the ROCK1/YAP1 pathway attenuated inflammation-induced NOX4 expression.

FIGURE S9. Targeting the ROCK1/YAP1 axis with fasudil and verteporfin reactivated the NRF2/HO-1 antioxidant pathway.

FIGURE S10. SIRT1 suppression was essential for the impairment of the NRF2/HO-1 antioxidant defense system induced by YAP1.

TABLE S1. The information of primary antibodies used in this study.

Antibodies	Sources	Information	Application
YAP1	Proteintech	Cat# 13584-1-AP; RRID: AB_2218915	WB 1:1000; IF 1:200
ROCK1	Proteintech	Cat# 21850-1-AP; RRID: AB_10953526	WB 1:1000; IF 1:200
CD4	Bioss	Cat# bs-0766R; RRID: AB_10857931	IF 1:200
CD8	Bioss	Cat# bs-4790R	IF 1:200
CD68	ServiceBio	Cat# GB113109, RRID:AB_2935658	IF 1:200
NOX4	Proteintech	Cat# 14347-1-AP; RRID: AB_10638146	IF 1:200
SIRT1	Affinity	Cat# DF6033, RRID:AB_2838007	WB 1:1000; IF 1:200
DRP1	Zenbio	Cat# 221099	WB 1:1000; IF 1:200
MFN2	Abcam	Cat# ab124773, RRID:AB_10999860	WB 1:1000; IF 1:200
NRF2	ABclonal	Cat# A1244; RRID: AB_2759282	WB 1:1000; IF 1:200
HO-1	Abcam	Cat# ab13248, RRID:AB_2118663	WB 1:1000; IF 1:200
PCNA	Boster	Cat# BM0104	IHC 1:400
α-SMA	Boster	Cat# BM0002	IHC 1:800
E-cadherin	Proteintech	Cat# 60335-1-Ig; RRID: AB_2881444	IHC 1:800; ICC 1:200
N-cadherin	Huabio	Cat# ET1607-37; RRID: AB_3069761	IHC 1:400
Vimentin	Abcam	Cat# ab92547; RRID: AB_10562134	ICC 1:200
COX-2	Abcam	Cat# ab179800; RRID: AB_2894871	WB 1:1000
VCAM-1	Abcam	Cat# ab134047; RRID: AB_2721053	WB 1:1000
HMGB1	Abmart	Cat# T55060; RRID: AB_2937051	WB 1:1000
BAX	Abmart	Cat# T40051; RRID: AB_2910262	WB 1:1000
BCL2	Abmart	Cat# T40056; RRID: AB_2929011	WB 1:1000
CYR61	ABclonal	Cat# A1111; RRID: AB_2758410	WB 1:1000
CTGF	ABclonal	Cat# A11067; RRID: AB_2758390	WB 1:1000
β-tubulin	Abmart	Cat# M20005; RRID: AB_2920648	WB 1:1000
GAPDH	Abmart	Cat# M20006; RRID: AB_2737054	WB 1:1000
Histone H3	Proteintech	Cat# 17168-1-AP; RRID: AB_2716755	WB 1:1000

TABLE S2. The sequences of RT-PCR primers used in this study.

**Gene (Rat)**	**Sequence (5'-3')**
Yap1 (F)	ACCCTCGTTTTGCCATGAAC
Yap1 (R)	TCCGTATTGCCTGCCGAAAT
Ccn1 (F)	AGAGGCTTCCTGTCTTTGGC
Ccn1 (R)	CTCGTGTGGAGATGCCAGTT
Ccn2 (F)	CTGACCTAGAGGAAAACATT
Ccn2 (R)	AGAAAGCTCAAACTTGACAG
Ankrd1 (F)	TTTCGAGTAGAGGAGCTGGTAAC
Ankrd1 (R)	CAGGTTCACACTGTTGGCTG
Birc5 (F)	CCACTGCCCTACCGAGAATG
Birc5 (R)	TGGTCTCCTTTGCAATTTTGTTCT
Amotl1 (F)	CGCTCCACCTTGTGAGGG
Amotl1 (R)	AGCGACTCACGAACAAGTCA
Tgfb1 (F)	CTGCTGACCCCCACTGATAC
Tgfb1 (R)	AGCCCTGTATTCCGTCTCCT
Acta2 (F)	CAGCTATGTGGGGGACGAAG
Acta2 (R)	TCCGTTAGCAAGGTCGGATG
Fn1 (F)	GATGAGCTTCCCCAACTGGT
Fn1 (R)	CTGGGTTGTTGGTGGGATGT
Lox (F)	CACACACACAGGGGTTGAGT
Lox (R)	CGTGATGTCCTGTGTAGCGA
Col1a1 (F)	ACCCCAAGGAGAAGAAGCAT
Col1a1 (R)	GGTCAGCTGGATAGCGACAT
Col3a1 (F)	AGAGGCTTTGATGGACGCAA
Col3a1 (R)	GGTCCAACCTCACCCTTAGC
Gapdh (F)	AGTGCCAGCCTCGTCTCATA
Gapdh (R)	GACTGTGCCGTTGAACTTGC

TABLE S3. Evaluating the prostatic pathological alterations using the Histoscore protocol.

**Histoscore**	**NC**	**YAP1-OE**	**P-value**
Low-power magnification (100×)			
Luminal shape: regular (1); villous (3); papillary (4);	2.37	3.77	<0.001
Acinar shape: tubular (1); branched (3); irregular (5)	2.40	3.73	<0.001
Interacinar space: large or moderate (1); back-to-back glands (5)	1.27	2.73	0.001
Stroma: fine (1); abundant (3); fibrosis/severe smooth muscle hyperplasia (5)	2.07	3.53	<0.001
High-power magnification (400×)			
Epithelial shape: flattened or cuboidal (1); cylindrical (3); hexagonal (5)	1.20	2.87	<0.001
Number of layers: mono-, 1 (1); oligo, 2-4 (3); pluri, ＞5 (5)	1.40	3.00	<0.001
If layer ＞1， then add: focal (3); diffuse (5)	0.60	3.90	<0.001
Alignment:			
Polar (1); apolar (3)	1.27	1.53	0.200
If there is piling up of epithelial cells, then add (3)	0.60	2.50	<0.001
If there is budding out of epithelial cells into stroma, then add (5)	0.17	2.33	<0.001
If periacinar clusters of epithelial cells are found, then add (3)	0.00	1.40	<0.001
If isolated clusters of epithelial cells are found outside acini, then add (5)	0.17	0.33	0.557
Lesion distribution			
Unilobar: isolated (2); multiple (6)	0.80	5.07	<0.001
Bilobar: isolated (4); multiple (8)	1.07	6.40	<0.001
Nuclear shape			
Round, regular (1); irregular (5)	1.27	1.93	0.073
Small or large (2); small and large in the same acinus (4)	2.13	2.57	0.053
Mitoses per field: absent, 0 (0); isolated, 1-2 (2); abundant, 3-5 (5); excessive, ＞5 (10)	1.13	3.37	<0.001
Basement membrane:			
Intact (1); interrupted (5)	1.27	2.47	0.005
Thin (1); thick (5)	1.93	2.87	0.060
Total score (Histoscore)	23.10	42.53	<0.001

Data were expressed as mean. The numbers in parentheses indicated the score assigned when the preceding description applies.

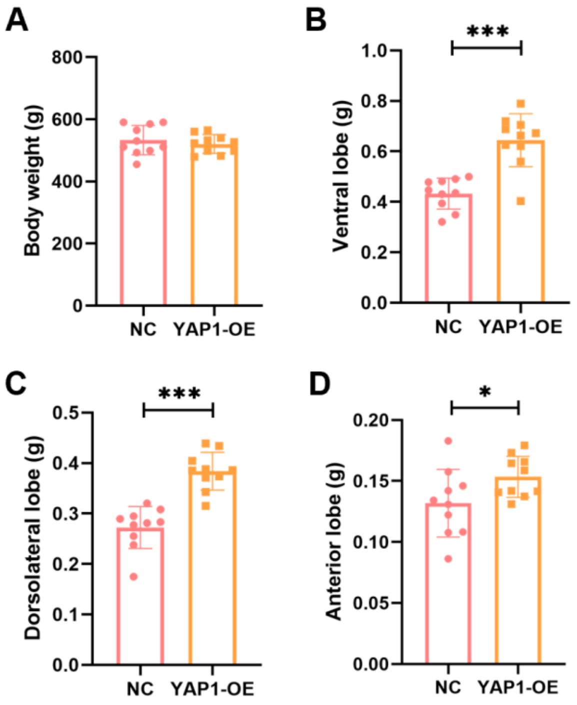

FIGURE S1. YAP1 overexpression induced diffuse enlargement of all prostate lobes in rats. (A) Rat body weight measured at the study endpoint. (B-D) Weights of the ventral, dorsolateral and anterior lobes of rat prostate.

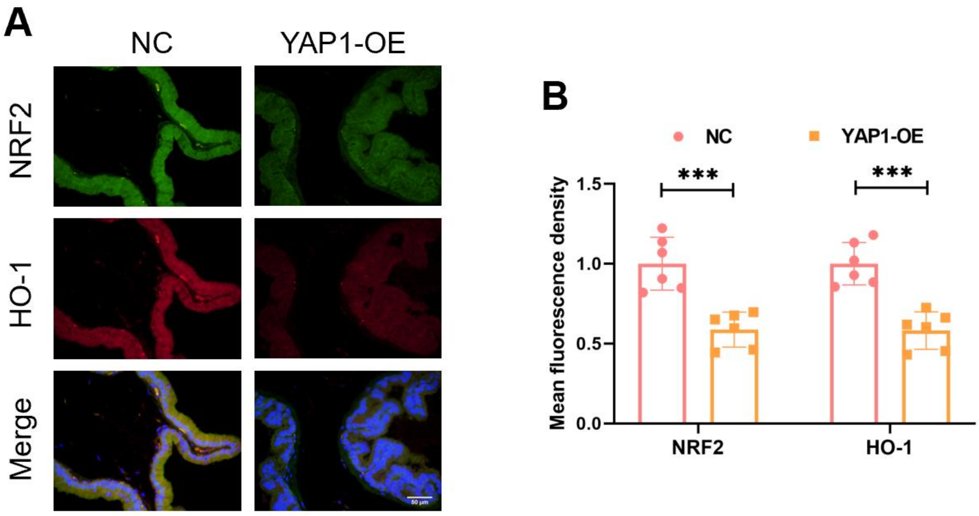

FIGURE S2. YAP1 overexpression suppressed NRF2/HO-1 antioxidant defense system. (A, B) Representative fluorescent images of NRF2 and HO-1 in the prostate of the two groups.

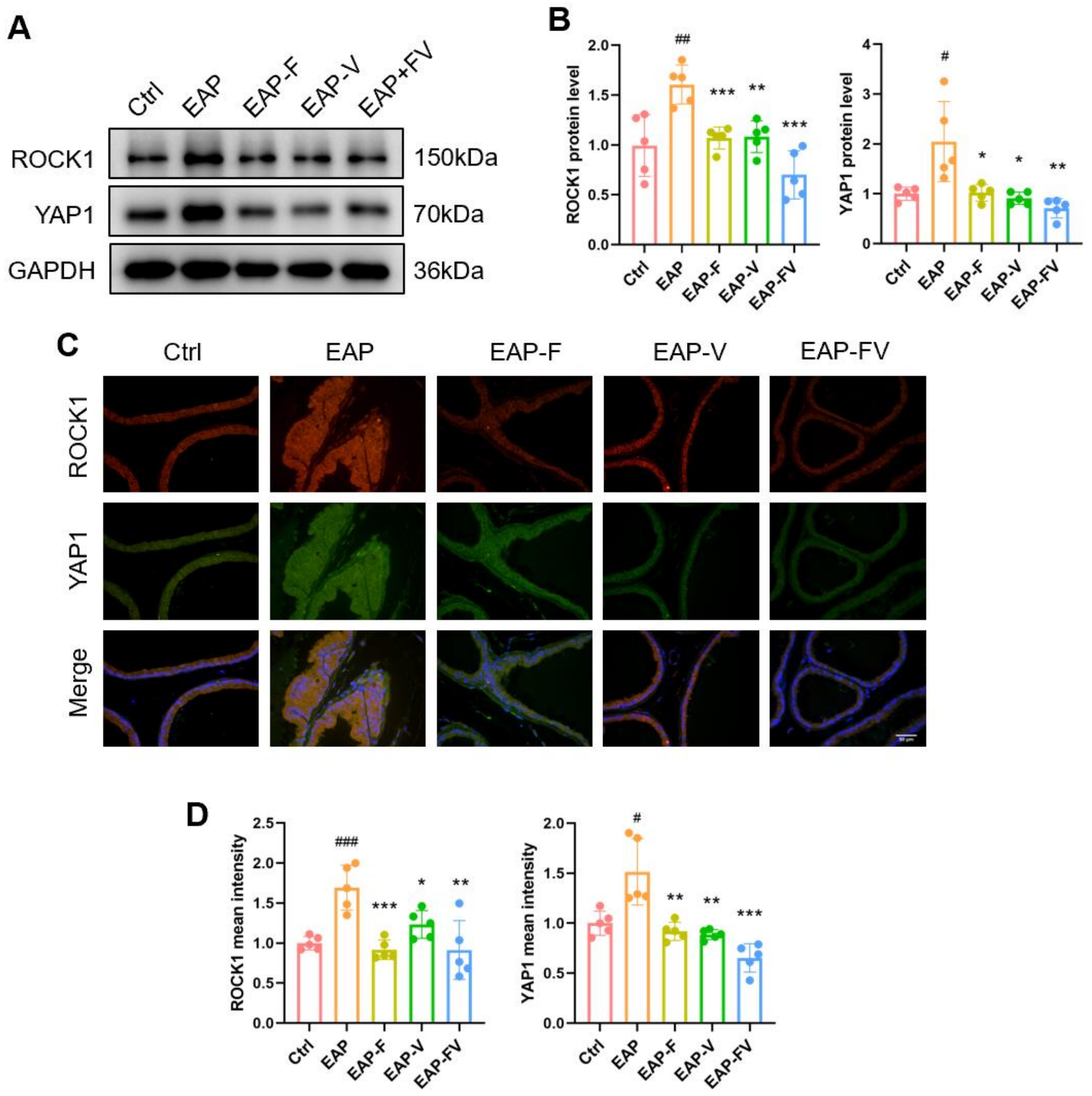

FIGURE S3. Fasudil and verteporfin were sufficient to inhibit inflammation-induced prostatic ROCK1 and YAP1 expression. (A, B) WB method confirming the inhibitory effects of fasudil and verteporfin on ROCK1/YAP1 expression. (C, D) Representative fluorescent images of ROCK1 and YAP1 in the prostate of different groups.

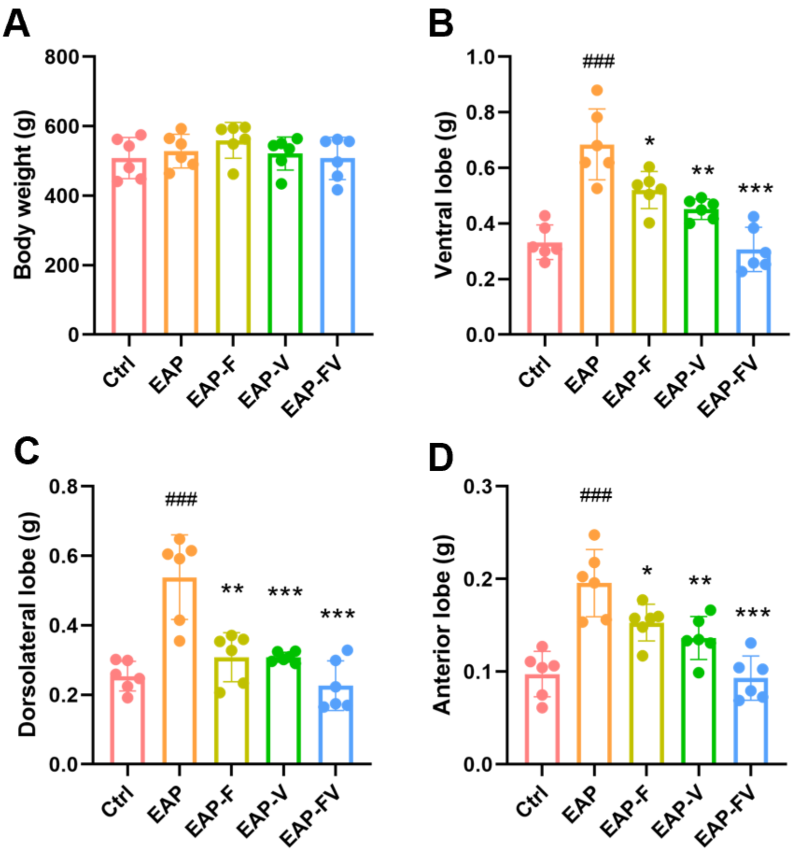

FIGURE S4. Targeting the ROCK1/YAP1 pathway attenuated inflammation-induced enlargement of various prostate lobes. (A) Final body weight of rats at study endpoint. (B-D) Mass of the ventral, dorsolateral and anterior prostate lobes.

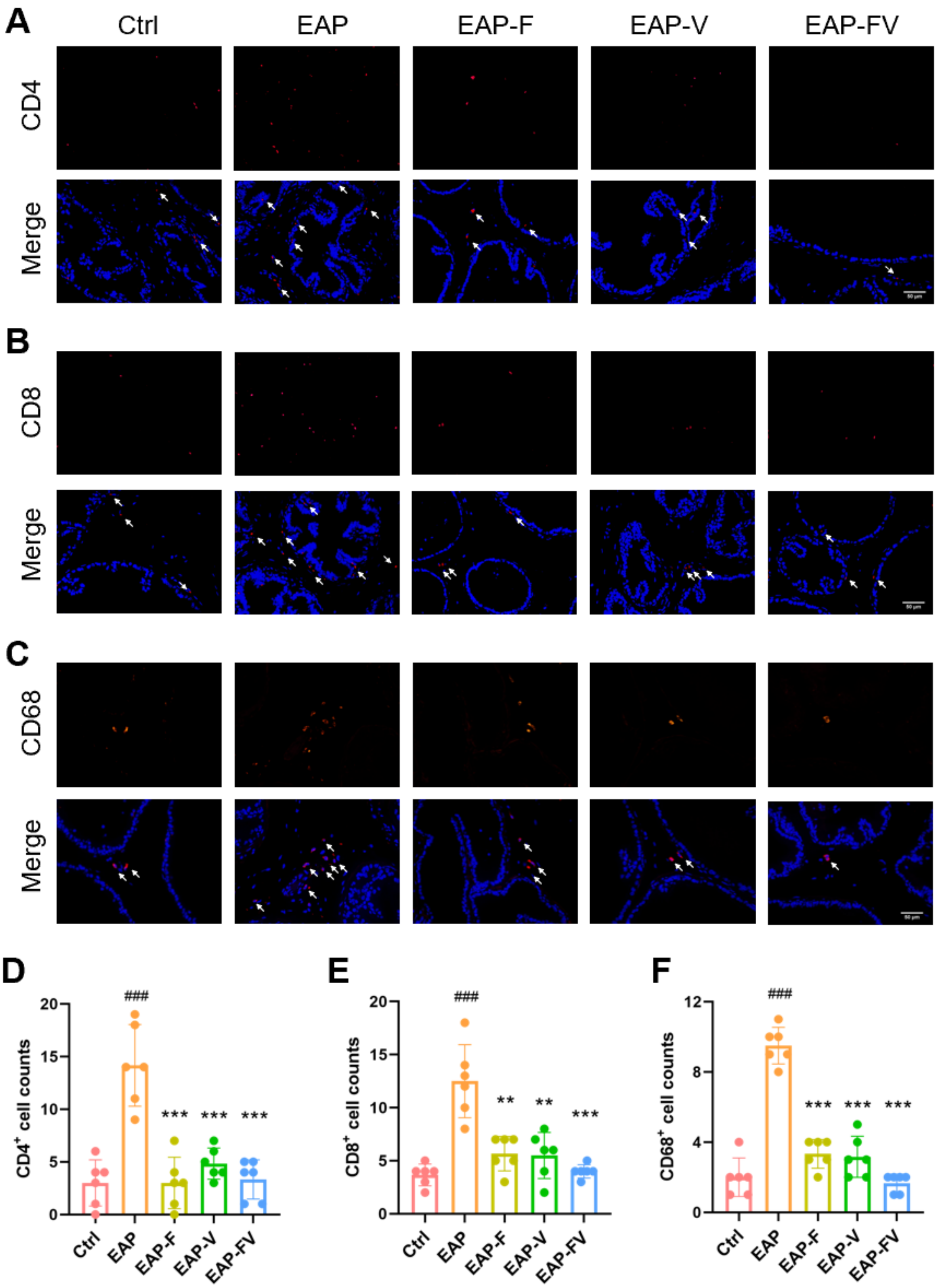

FIGURE S5. Inhibition of the ROCK1/YAP1 axis prevented inflammatory cells infiltration in prostate. (A-C) IF analysis illustrating CD4^{+} T helper cells, CD8^{+} T cytotoxic cells and CD68^{+} macrophages. (D-F) The positive cells were counted and compared among different groups.

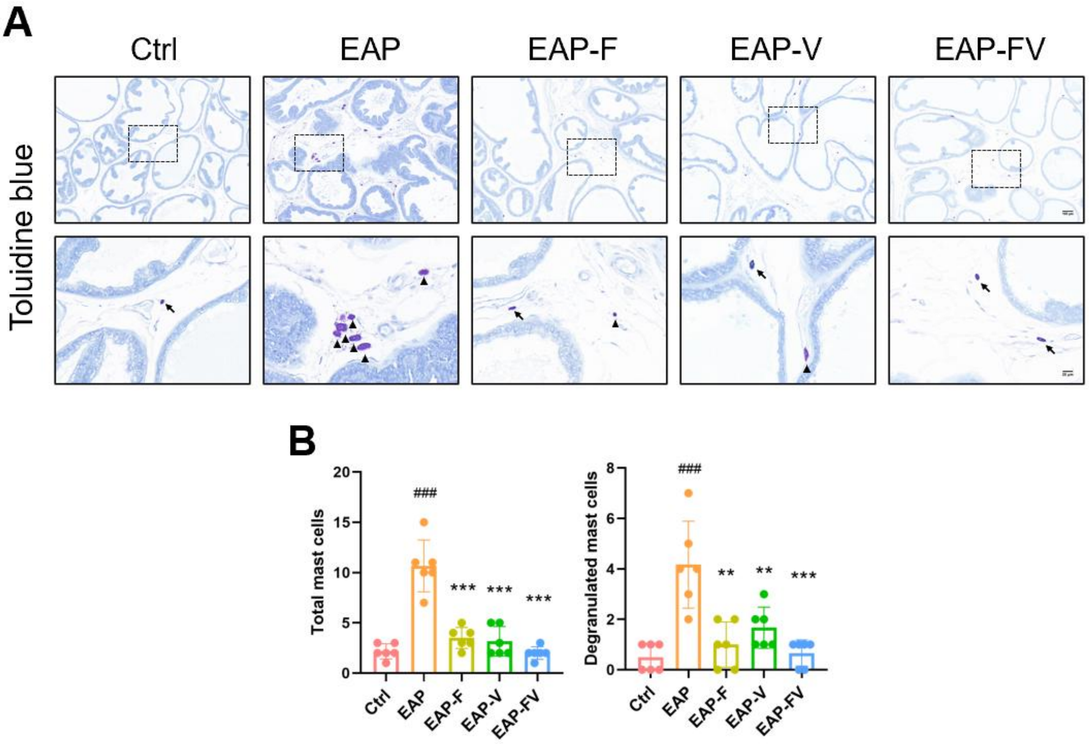

FIGURE S6. Inhibition of the ROCK1/YAP1 axis decreased mast cell accumulation and activation. (A, B) Toluidine blue staining was used to identify mast cells in different groups. Arrows denote the quiescent mast cells with intact cytomembranes, while triangles indicate the degranulated mast cells with dispersing metachromatic granules.

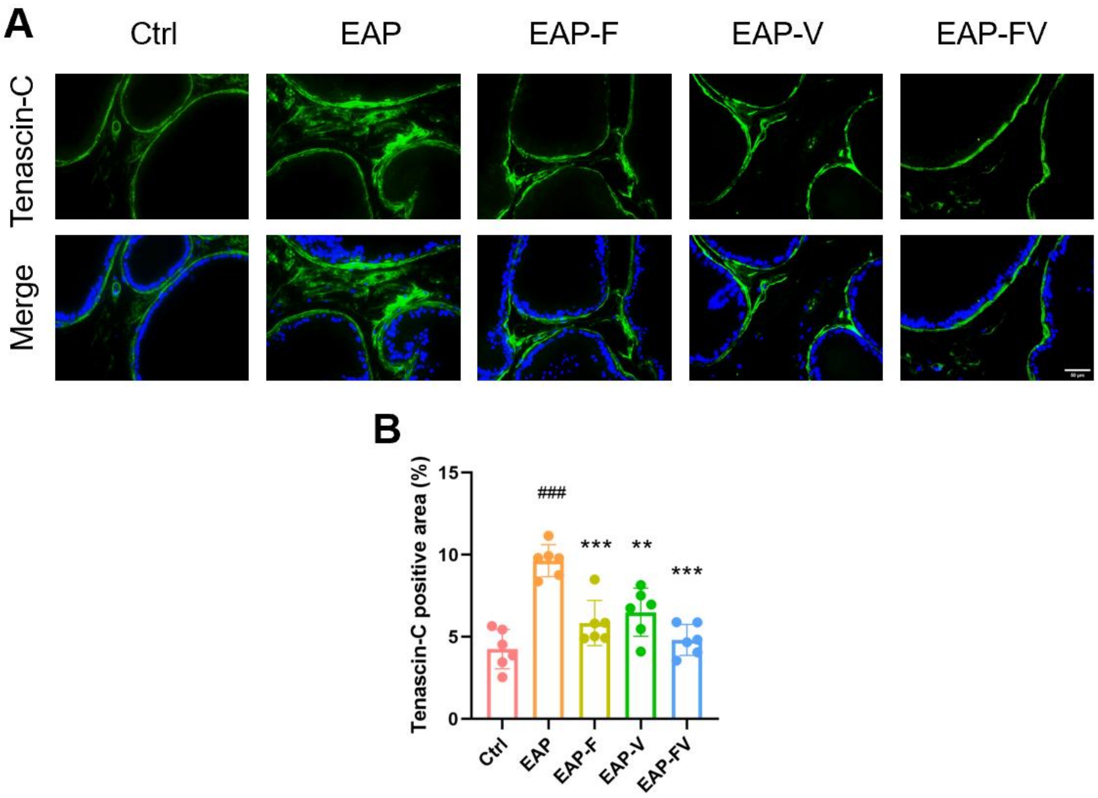

FIGURE S7. Inhibition of the ROCK1/YAP1 pathway reduced inflammation-induced Tenascin-C expression. (A, B) Representative fluorescence images illustrating Tenascin-C levels in prostatic tissues across groups.

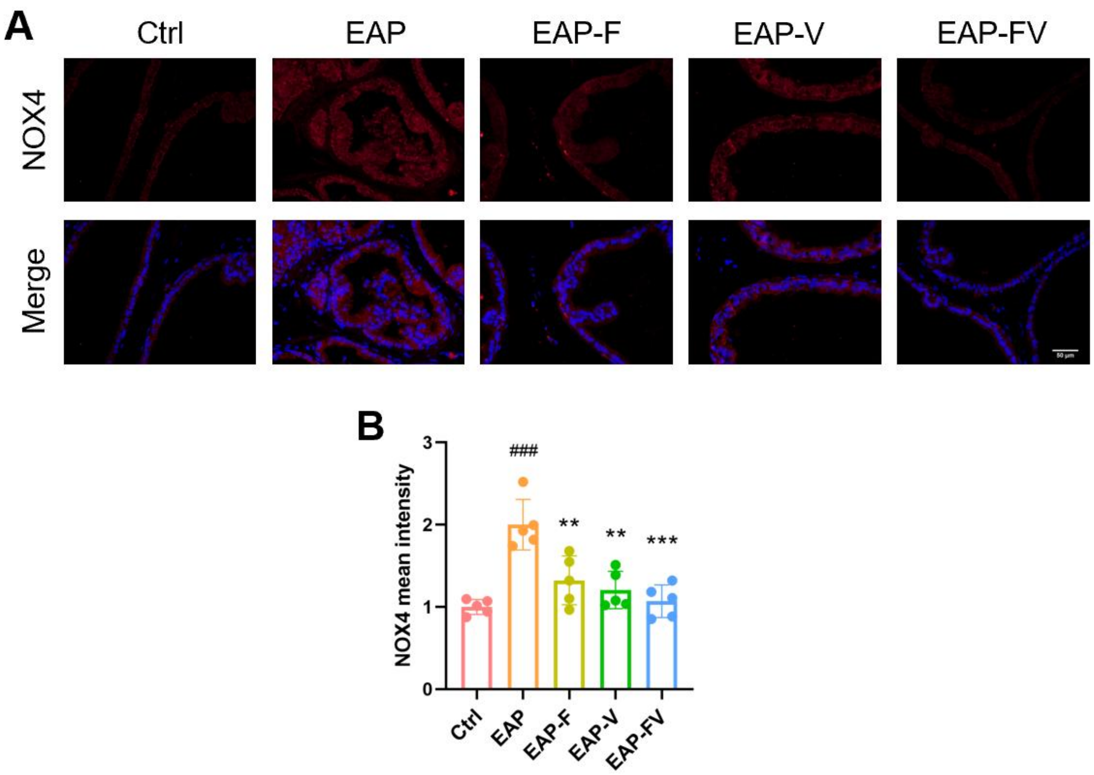

FIGURE S8. Suppression of the ROCK1/YAP1 pathway attenuated inflammation-induced NOX4 expression. (A, B) Representative fluorescence images showing the expression of NOX4 in the prostate of each group.

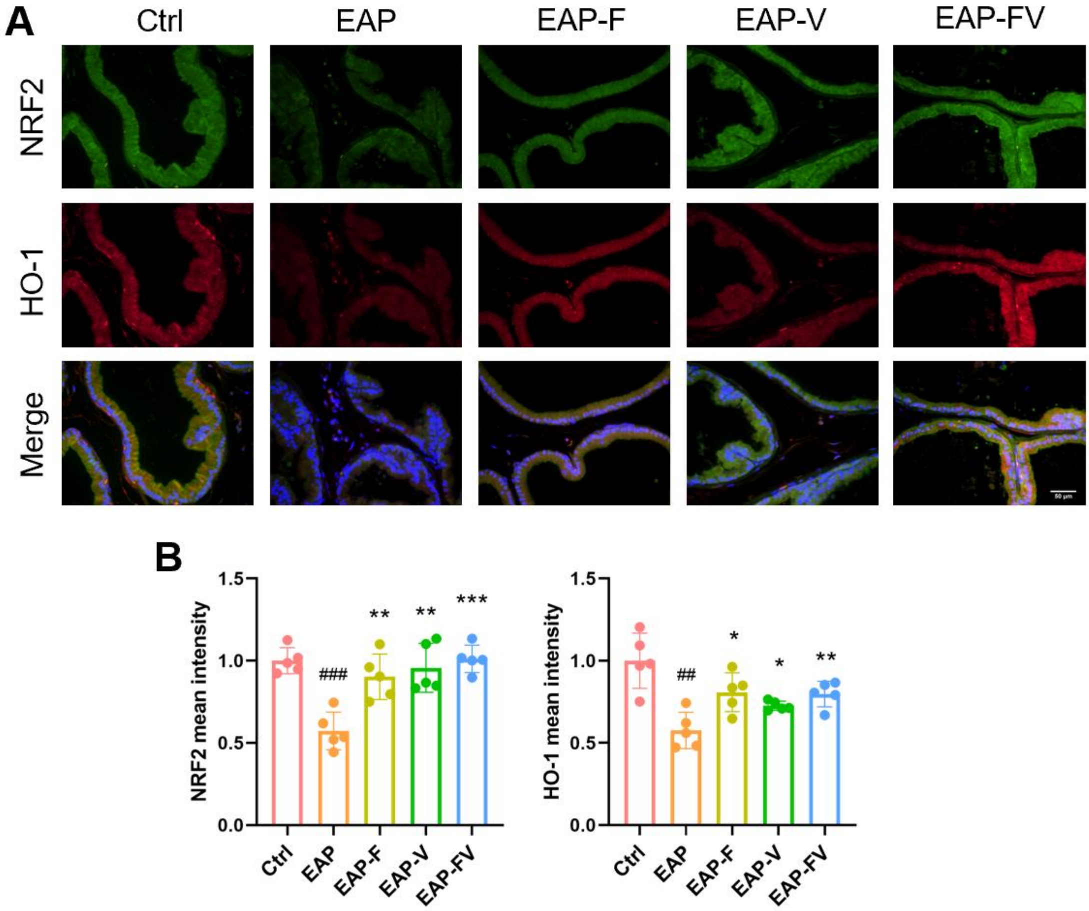

FIGURE S9. Targeting the ROCK1/YAP1 axis with fasudil and verteporfin reactivated the NRF2/HO-1 antioxidant pathway. (A, B) Representative fluorescence images to determine the expression of NRF2 and HO-1 in the prostate of each group.

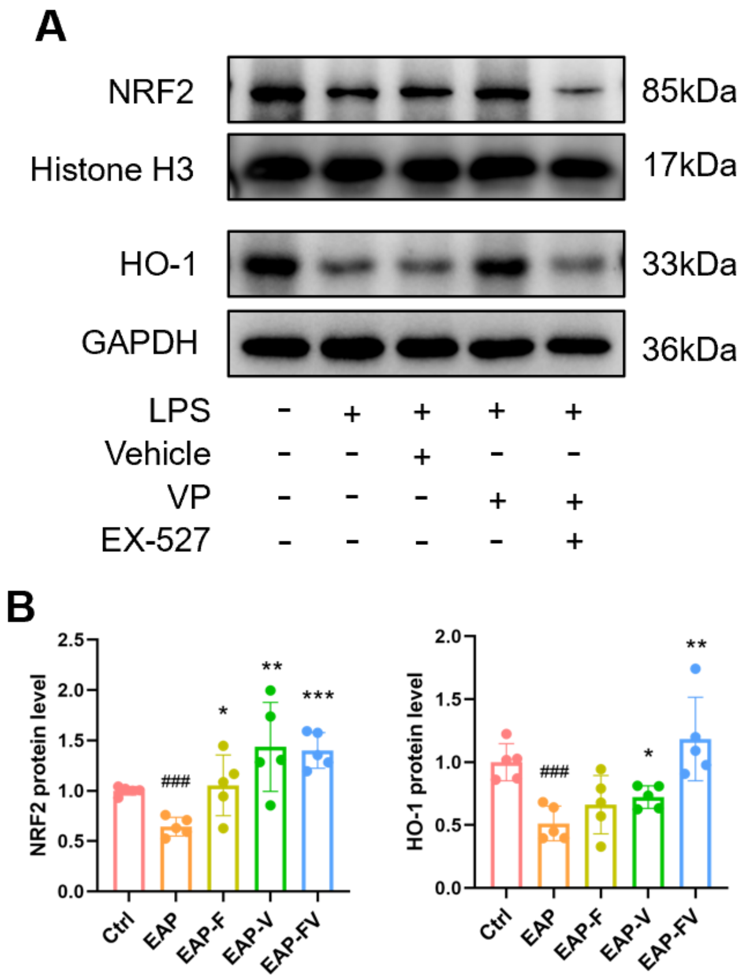

FIGURE S10. SIRT1 suppression was essential for the impairment of the NRF2/HO-1 antioxidant defense system induced by YAP1. (A, B) WB analysis to evaluate the effects of the YAP1 inhibitor VP and the SIRT1 inhibitor EX-527 on the expression of NRF2 and HO-1 in the LPS-stimulated BPH-1 cells.
